# The Neuroprotective Mechanisms of PPAR‐γ: Inhibition of Microglia‐Mediated Neuroinflammation and Oxidative Stress in a Neonatal Mouse Model of Hypoxic‐Ischemic White Matter Injury

**DOI:** 10.1111/cns.70081

**Published:** 2024-11-04

**Authors:** Mingchu Fang, Qianqian Yu, Jiahao Ou, Jia Lou, Jianghu Zhu, Zhenlang Lin

**Affiliations:** ^1^ Department of Neonatology The Second Affiliated Hospital and Yuying Children's Hospital of Wenzhou Medical University Wenzhou Zhejiang China; ^2^ The Second School of Medicine Wenzhou Medical University Wenzhou Zhejiang China; ^3^ Key Laboratory of Perinatal Medicine of Wenzhou Wenzhou Zhejiang China; ^4^ Key Laboratory of Structural Malformations in Children of Zhejiang Province Wenzhou Zhejiang China

**Keywords:** microglia, neuroinflammation, oxidative stress, PPAR‐γ, white matter injury

## Abstract

**Background:**

Neuroinflammation and oxidative stress, mediated by microglial activation, hinder the development of oligodendrocytes (OLs) and delay myelination in preterm infants, leading to white matter injury (WMI) and long‐term neurodevelopmental sequelae. Peroxisome proliferator‐activated receptor gamma (PPAR‐γ) has been reported to inhibit inflammation and oxidative stress via modulating microglial polarization in various central nervous system diseases. However, the relationship between PPAR‐γ and microglial polarization in neonatal WMI is not well understood. Therefore, this study aimed to elucidate the role and mechanisms of PPAR‐γ in preterm infants affected by WMI.

**Methods:**

In this study, an in vivo hypoxia‐ischemia (HI) induced brain WMI neonatal mouse model was established. The mice were administered intraperitoneally with either RSGI or GW9662 to activate or inhibit PPAR‐γ, respectively. Additionally, an in vitro oxygen–glucose deprivation (OGD) cell model was established and pretreated with pcDNA 3.1‐PPAR‐γ or si‐PPAR‐γ to overexpress or silence PPAR‐γ, respectively. The neuroprotective effects of PPAR‐γ were investigated in vivo. Firstly, open field test, novel object recognization test, and beam‐walking test were employed to assess the effects of PPAR‐γ on neurobehavioral recovery. Furthermore, assessment of OLs loss and OL‐maturation disorder, the number of myelinated axons, myelin thickness, synaptic deficit, activation of microglia and astrocyte, and blood–brain barrier (BBB) were used to evaluate the effects of PPAR‐γ on pathological repair. The mechanisms of PPAR‐γ were explored both in vivo and in vitro. Assessment of microglia polarization, inflammatory mediators, reactive oxygen species (ROS), MDA, and antioxidant enzymes was used to evaluate the anti‐inflammatory and antioxidative effects of PPAR‐γ activation. An assessment of HMGB1/NF‐κB and NRF2/KEAP1 signaling pathway was conducted to clarify the mechanisms by which PPAR‐γ influences HI‐induced WMI in neonatal mice.

**Results:**

Activation of PPAR‐γ using RSGI significantly mitigated BBB disruption, promoted M2 polarization of microglia, inhibited activation of microglia and astrocytes, promoted OLs development, and enhanced myelination in HI‐induced WMI. Conversely, inhibition of PPAR‐γ using GW9662 further exacerbated the pathologic hallmark of WMI. Neurobehavioral tests revealed that neurological deficits were ameliorated by RSGI, while further aggravated by GW91662. In addition, activation of PPAR‐γ significantly alleviated neuroinflammation and oxidative stress by suppressing HMGB1/NF‐κB signaling pathway and activating NRF2 signaling pathway both in vivo and in vitro. Conversely, inhibition of PPAR‐γ further exacerbated HI or OGD‐induced neuroinflammation, oxidative stress via modulation of the same signaling pathway.

**Conclusions:**

Our findings suggest that PPAR‐γ regulates microglial activation/polarization as well as subsequent neuroinflammation/oxidative stress via the HMGB1/NF‐κB and NRF2/KEAP1 signaling pathway, thereby contributing to neuroprotection and amelioration of HI‐induced WMI in neonatal mice.

## Graphical abstract

Our findings suggest that PPAR‐γ regulates microglial activation/polarization as well as subsequent neuroinflammation/oxidative stress via the HMGB1/NF‐κB and NRF2 signaling pathway, thereby contributing to myelination and amelioration of WMI after HI insult in neonatal mice.

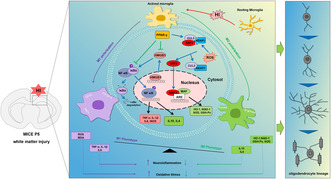

## 1 Introduction

Advancements in perinatal and neonatal care have significantly improved the survival rate of critical preterm infants and reduced complications. However, the occurrence of brain injury and life‐long sequelae in survivors of premature birth remains unchanged [1]. The EPIPAGE‐2 cohort study revealed that 27.8%, 18.7%, and 11.6% of children born at 24–26, 27–31, and 32–34 weeks gestational age, respectively, exhibited severe or moderate neurodevelopmental disabilities at age 5 [2]. Peri‐ and postnatal white matter injury (WMI) is the major form of brain injury which affects approximately 42.5% of premature birth survivors [3, 4], leading to the arrested development of the oligodendrocytes (OLs) and myelin sheath dysplasia due to pre‐OL vulnerability [5, 6, 7]. WMI has been linked to motor disabilities, cognitive deficits, neurosensory impairments, and behavioral issues in children and juveniles [8, 9]. Unfortunately, there are no effective treatments available for promoting neurofunctional recovery in the increasing population of preterm infants with WMI [10]. Therefore, the development of innovative neuroprotective strategies to combat WMI, validated in clinically relevant animal models, is urgently needed.

Peri‐ and postnatal insults, such as cerebral hypoxia‐ischemia (HI), and inflammatory reactions contribute to WMI in preterm infants [5]. Notably, HI is a significant risk factor for WMI. Studies indicate that perinatal WMI is primarily driven by inflammatory responses, oxidative stress, cytokine toxicity, and glutamate excitotoxicity [11]. Among them, inflammation and oxidative stress are the predominant causes of WMI in premature infants [12, 13]. Microglial activation and polarization to an M1 phenotype triggered by HI can lead to neuroinflammatory responses and excessive oxidative stress, negatively impacting the development of OL lineage cells and resulting in decreased myelin sheath formation [14, 15, 16, 17]. Therefore, modulating microglial activation and polarization is a promising strategy for mitigating impairments associated with HI‐induced WMI in preterm infants.

Peroxisome proliferator‐activated receptor gamma (PPAR‐γ), a ligand‐responsive nuclear transcription factor classified within the type II nuclear hormone receptor superfamily, is integral to a variety of pathophysiological mechanisms. Previously, PPAR‐γ is identified as an important regulator of glucose homeostasis and subcutaneous fat formation [18]. Furthermore, as recently extensively reviewed, PPAR‐γ also regulates oxidative stress [19], attenuates inflammation [19, 20], inhibits neuronal apoptosis [21], promotes cell proliferation [22], and maintains vascular endothelial function [23] in brain injury and repair. PPAR‐γ directly interacts with transcription factor (nuclear transcription factor‐κB, NF‐κB) and regulates activation of nuclear factor‐E2‐related factor 2 (NRF2), leading to the suppression of inflammation and oxidative stress in microglial activation [24]. Notably, later works on PPAR‐γ associated with ischemic brain injury reveal that its influence on microglial activation and polarization is primarily attributed to its role in regulating antioxidative processes and inflammatory response [25, 26]. The precise role of PPAR‐γ in regulating microglial activation and polarization and mitigating neuroinflammation and oxidative stress in neonatal hypoxic‐ischemic WMI remains to be elucidated, along with the associated specific mechanisms.

In this study, we investigated the neuroprotective effects of PPAR‐γ on WMI and neurological disabilities using a mouse model of neonatal HI‐induced WMI. We also explored the molecular mechanisms underlying the effects of PPAR‐γ on microglial activation and polarization both in vivo and in vitro. Our findings suggest that PPAR‐γ emerges as a potential therapeutic target for WMI in preterm infants.

## 2 Materials and Methods

### 2.1 Animals and Ethical Approval

Animal procedures were conducted in strict compliance with the guidelines set forth by the Animal Ethics Committee of Wenzhou Medical University and the National Institutes of Health Guide for the Care and Use of Laboratory Animals. C57Bl/6J male and female mice (6 to 8 weeks old) were obtained from Vital River (Zhejiang, China) and bred under standard laboratory conditions with ad libitum access to water and fodder, a 12 h light‐cycle, as well as temperature‐ and humidity‐controlled environments. The pups were generated by crossing male and female mice. Male pups at postnatal day 5 (P5) were utilized, corresponding to 23–32 weeks of gestation in terms of OL development in the human brain [27]. The animal experiments strictly adhered to the principles of replacement, reduction, and refinement (3Rs).

### 2.2 Neonatal Mice WMI Model and Treatment

The neonatal WMI model was established following previously described methods [27]. Briefly, P5 pups underwent anesthesia using isoflurane inhalation. The left common carotid artery was carefully ligated between double ligatures after separation before being severed; subsequently, the wound was sutured. The total operation time did not exceed 4 min. Following this procedure, mice were allowed to recover for 1.5 h with their mother before being placed in a temperature‐controlled hypoxic container (36.0°C) with humidified mixed gas (10% oxygen and 90% nitrogen at a flow rate of 2 L/min) for 60 min. Sham‐operated mice without exposure to hypoxia constituted the Sham group. Animals that succumbed during these procedures (mortality rate: 5%) were excluded from further experimentation.

**FIGURE 1:**
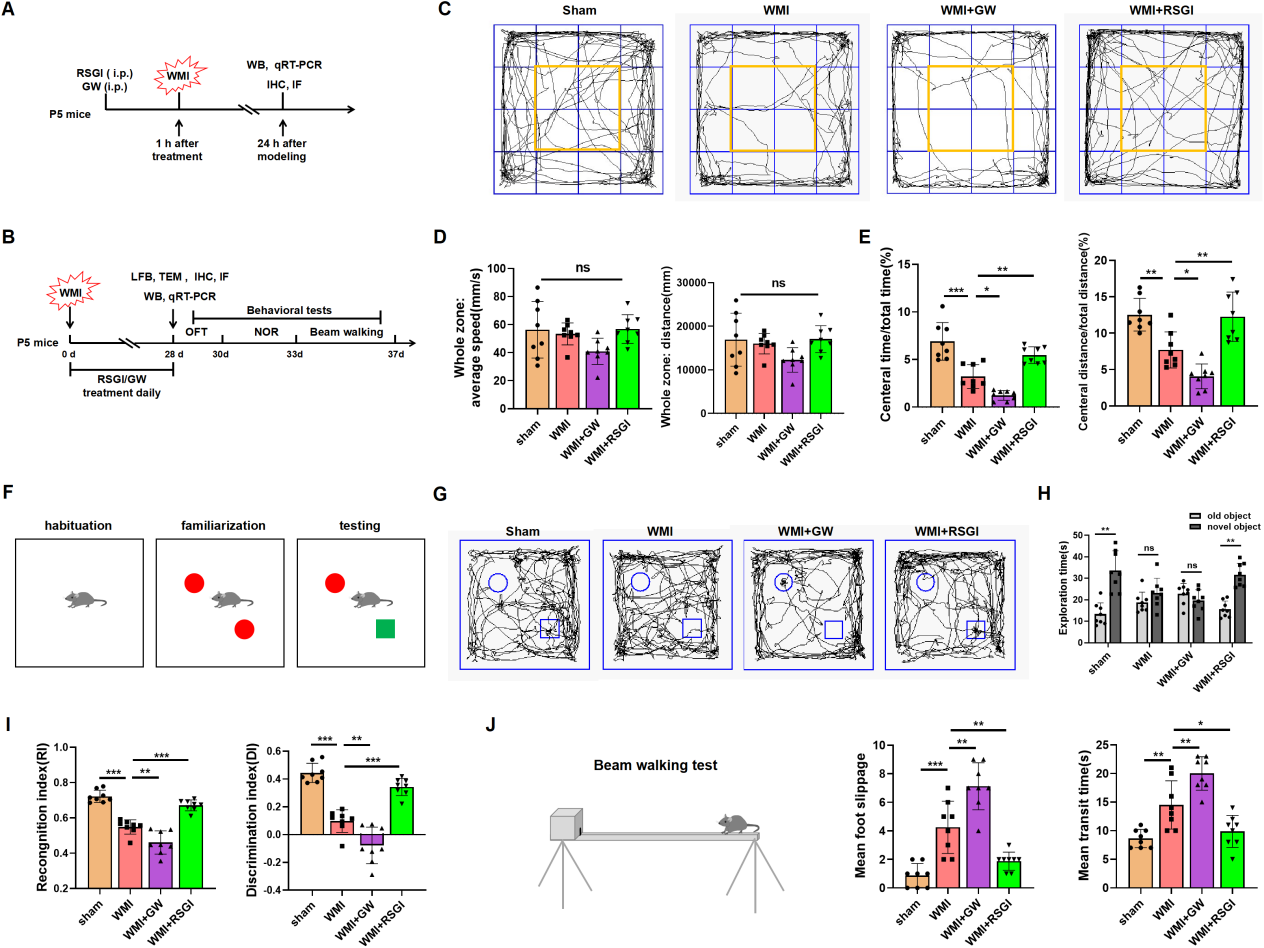
Effects of PPAR‐γ on neurodeficits in the neonatal WMI model. (A, B) Diagram of the in vivo experimental design. (C) OFT was conducted to evaluate anxiety‐like behavior, with representative images of route traces displayed. (D, E) The behaviors of the mice, such as total distance traveled, time spent in the central area, and distance traveled in the central area, were recorded and analyzed. (F, G) NOR test was performed to assess cognitive performance, featuring a test diagram and representative trajectory images. (H, I) Exploration time of the novel object, DI, and RI during testing phase were recorded and analyzed. (J) Beam‐walking test was employed to evaluate motor coordination and balance, with hind limb slippage frequency and time to traverse beam recorded and analyzed. Graph displays mean ± SD values (*n* = 8 mice per group). *p < 0.05, **p < 0.01, and ***p < 0.001, significance based on one‐way ANOVA with Tukey's post hoc test.

The mice were allocated into four groups using a randomized block design: sham group, WMI group, WMI + rosiglitazone (WMI + RSGI) group, and WMI + GW9662 (WMI + GW) group. Rosiglitazone (BRL 49653, MCE, Shanghai, China) is a widely utilized PPAR‐γ agonist while GW9662 (HY‐16578, MCE, Shanghai, China) serves as an efficient PPAR‐γ antagonist. Both compounds were dissolved in DMSO, with the final concentration of DMSO being 1% in the solutions diluted with phosphate‐buffered saline (PBS). The drug delivery method and dosages used in this study were based on preliminary investigations [28, 29, 30]. In the WMI + RSGI group, mice received intraperitoneal injections of Rosiglitazone at a dose of 6 mg/kg 1 h before establishing the WMI model and once daily for 28 days. For the WMI + GW group, mice were administered GW9662 (1 mg/kg) via intraperitoneal injection 1 h before modeling daily for 28 days. The sham and WMI groups of mice received equivalent volumes of 1% DMSO through intraperitoneal injections performed daily for 28 days. The diagram of the in vivo experimental design is shown in Figure 1A,B.

### 2.3 Cell Culture and Oxygen–Glucose Deprivation (OGD) Model

The human microglial clone three (HMC3) cell line was procured from Shanghai FuHeng Biology located in Shanghai, China. The cells were cultured optimally using Dulbecco's modified eagle medium (DMEM) supplemented with 10% fetal bovine serum (FBS) and 1% penicillin–streptomycin (P/S) (Gibco, United States). They underwent incubation within a humidified environment at 37°C supplied with a 5% CO_2_. OGD induction followed previous protocols but included modifications. HMC3 cells were seeded in 6‐well plates and allowed to adhere and grow for 24 h. The next day, the cells were rinsed twice with PBS and subsequently placed under serum/glucose‐free DMEM condition within an anoxic incubator (Thermo Fisher Scientific) set at 1% oxygen concentration alongside 5% CO_2_ to stimulate HI conditions in vitro. Control group cells underwent similar treatment without exposure to OGD.

### 2.4 Cell Counting Kit‐8 Assay

To determine the appropriate duration of OGD for the in vitro experiments, we performed a cell viability assay on HMC3 cells utilizing Cell Counting Kit‐8 (CCK‐8, 40203ES88, Yeasen Biotechnology, Shanghai, China). Cells were seeded at a density of 1 × 10^4^ cells/mL in 100 μL culture medium per well into 96‐well plates and incubated for 24 h. Subsequently, cells were exposed to OGD for varying durations including 6 h, 12 h, 18 h, and 24 h. After OGD, the culture medium was removed, and CCK‐8 solution (1:10) diluted in fresh medium was added into each well at an equivalent volume of 100 μL per well. Following a 2‐h incubation in darkness at 37°C, the absorbance was then measured at 450 nm using a microplate reader (Thermo Fisher Scientific, Waltham, MA, United States).

### 2.5 Short Interfering (Si) RNA and Plasmid Transfection

The PPAR‐γ siRNA (si‐PPAR‐γ) and PPAR‐γ overexpression plasmid vector (pcDNA3.1‐PPAR‐γ) utilized in the experiments were synthesized by RiboBio Co. Ltd. (Guangzhou, China). HMC3 cells were seeded in 6‐well plates and allowed to stabilize for 24 h. Subsequently, transfection with si‐PPAR‐γ or pcDNA3.1‐PPAR‐γ was carried out using Lipofectamine 3000 Transfection Kit (Invitrogen, Carlsbad, CA, USA) following the manufacturer's instructions. At 48 h after transfection, the cells were exposed to OGD for 18 h and then collected for subsequent experiments.

### 2.6 Reactive Oxygen Species (ROS) Detection

An ROS Assay Kit (S0033S, Beyotime, Shanghai, China) was employed to detect intracellular ROS as per established protocols. Following various treatments, HMC3 cells were washed with fresh medium and incubated with DCFH‐DA (10 μM) in fresh medium for 30 min in the dark. The cells were then rinsed thrice with fresh medium and counterstained with Hoechst at 37°C for 10 min. Images were captured using a fluorescence inverted microscope (Ni‐U, Nikon, Japan), and the fluorescence intensities of DCFH‐DA were quantified using Image J software.

### 2.7 Open Field Test (OFT)

Anxiety‐related behavior was assessed through an open field test conducted 28 days after HI injury. Mice were individually placed in an acrylic glass box measuring 40 × 40 cm, and their movement trajectories were automatically tracked for a duration of 5 min [31]. The central area of the box was defined as a field measuring dimensions of 20 × 20 cm located at its center. The behaviors exhibited by mice including total distance traveled, the percentage of time spent in the central area, and the percentage of distance traveled in the central area were analyzed using animal behavioral analysis system (Shanghai JiLiang SoftwareTechnology Co. Ltd., Shanghai, China).

### 2.8 Novel Object Recognition (NOR) Test

As previously described [32], the task was conducted from 30 to 32 days after WMI establishment. The NOR test consisted of three stages: habituation, object familiarization, and NOR testing. During the habituation stage on the first day, mice were acclimated in an open field box (40 cm in height, 25 cm in width, 25 cm in length) for 5 min. On the second day during object familiarization, mice had 5 min to explore two identical objects in a familiar box before being returned to their cage for 1 h. In the testing phase, one of the previously presented objects was replaced with a novel object and mice were given 5 min to explore both objects. Exploration was recorded when mice directed their nose within a distance < 1 cm from the object and/or touched it with their nose. The movement trajectory and exploration time toward each object were analyzed using ANYmaze 7.0 (IL, USA). The Recognition Index (RI) is determined by the formula (exploration time of novel object/exploration time of both objects) × 100, while the Discrimination Index (DI) is calculated as [(exploration time of novel object—exploration time of old object)/exploration time of both objects] × 100.

### 2.9 Beam‐Walking Test

The beam‐walking task was conducted 33 days after WMI establishment as previously reported [32], with modification. A 0.5 cm‐wide beam was positioned 50 cm above the ground, with a black box placed at the beam's end as a target for the mouse to reach. During training, padding material from their own cages was placed in the box to attract them to walk through the beam. Mice received three trials per day for 3 days prior to assessment, and performance was observed by recording videos while assessing motor coordination and balance based on hind limb slippage frequency and time taken to cross the beam.

### 2.10 Brain Water Content

Mice underwent deep anesthesia followed by decapitation. The left hemisphere was rapidly harvested and weighed to acquire the wet weight. The brain tissue was placed into an electrothermal oven at 70°C for 72 h to obtain the dry weight. The percentage of moisture content was calculated as [(Wet weight—Dry weight)/Wet weight] × 100%.

### 2.11 Measurement of GSH and MDA Levels

Mice underwent deep anesthesia followed by decapitation. White matter (WM) was promptly extracted, quantified, and homogenized in extraction buffer. HMC3 cells were collected and ultrasonically lysed. Malondialdehyde (MDA) and glutathione peroxidase (GSH‐Px) levels were then quantified using commercially available kits from Beyotime Technology (Shanghai, China) in accordance with the manufacturer's guidelines.

### 2.12 RT‐qPCR Analysis

The total RNA extracted from WM tissues and HMC3 cells at a specific time point using the TRIzol/chloroform method was subjected to RT–qPCR analysis. Equal amounts of RNA (1 μg) were reverse‐transcribed into cDNA utilizing the PrimeScript RT Master Mix kit (Takara Bio Inc., Japan) according to the manufacturer's protocol. PCR assays were carried out on the CFX96 Real‐Time PCR System (Bio‐Rad, Hercules, CA, USA) employing the TB Green Premix Ex Taq II kit (Takara Bio Inc., Japan). The mRNA expression level of the target gene was quantified using the equation $2^{-\Delta \Delta C_{t}}$ and normalized to β‐actin control. The primers utilized in this investigation were designed with the NCBI Primer‐Blast Tool and are detailed in Tables S1 and S2.

### 2.13 Western Blot Analysis

Mice were euthanized by intraperitoneal injection of an lethal dose of pentobarbital sodium at a specific time point. Brains were harvested on the ice‐cold medium, and WM tissues were isolated under a dissecting microscope as previously described. Samples of WM or HMC3 cells were lysed in ice‐cold RIPA lysis buffer supplemented with phenylmethane‐sulfonyl fluoride and phosphatase inhibitors (all from Solarbio, Beijing, China). The extraction of nuclear proteins followed standard procedures utilizing a Nuclear Proteins Extraction Kit (R0050; Solarbio Biotechnology). Protein concentrations were determined using the BCA protein assay kit (ZJ102L, Epizyme Biotech, Shanghai, China). Equal amounts of protein (30 μg for in vitro studies and 60 μg for in vivo studies) underwent separation via 10%–12% SDS‐PAGE gels before being transferred onto polyvinylidene fluoride membranes (Millipore, Billerica, MA, USA) using the Bio‐Rad wet‐blot system along with blot transfer buffer (25 mM Tris and 190 mM glycine) containing 20% methanol.

Following blocking with 5% bovine serum albumin (BSA) in Tris‐buffered saline with Tween 20 (TBST) for 2 h at room temperature, the membranes underwent three washes in TBST before being immunoblotted with specified primary antibodies (listed in Table S3) overnight at 4°C on a rotating shaker. After further washing steps, membranes were incubated with the appropriate HRP‐conjugated secondary antibodies: Anti‐Rabbit IgG (1:10,000, Proteintech, SA00001‐2), Anti‐Mouse IgG (1:10,000, Proteintech, SA00001‐1), or Anti‐goat IgG (1:5000, Proteintech, SA00001‐4) secondary antibodies at room temperature for 2 h. Proteins were visualized with the enhanced chemiluminescence (ECL) substrate (P2300, NCM Biotech, Suzhou, China) and imaged utilizing the ChemiDoc XRS Biorad Imager. Finally, densitometric measurements concluded through Image Lab Software5.1 (Bio‐Rad, CA, USA).

### 2.14 Luxol Fast Blue (LFB) Staining

Mice were humanely euthanized with a lethal dose of pentobarbital sodium via intraperitoneal injection and subsequently underwent transcardial perfusion with cold normal saline followed by 4% paraformaldehyde (PFA) in PBS at 28 days post‐induction of the WMI model. The brains were meticulously dissected, fixed in 4% PFA, dehydrated, and embedded in paraffin. Paraffin‐embedded brain samples were sectioned coronally into 5 μm‐thick slices using a HistoCore Biocut (Leica, Weztlar, Germany) and mounted on slides. The paraffin sections were stained with Luxol Fast Blue (LFB) staining (Solarbio Biotechnology) according to the manufacturer's instructions. Bright‐field images were acquired using an optical microscope (Nikon, Tokyo, Japan) and analyzed using image analysis software (ImageJ).

### 2.15 Immunohistochemistry

The coronal tissue sections were prepared as previously described. Following deparaffinization in xylene and rehydration in an ethanol gradient, the sections were treated with 3% hydrogen peroxide at room temperature for 15 min. Antigen retrieval was achieved by heating the sections in preheated sodium citrate buffer for 25 min. After cooling and three washes in PBS, the sections were blocked with a solution containing 10% normal goat serum and 0.3% Triton X‐100 for 1 h at room temperature. Incubation with primary antibodies (listed in Table S3) was carried out overnight at 4°C. Subsequently, the sections were incubated with a goat anti‐rabbit IgG horseradish peroxidase‐labeled secondary antibody (1:200) for 2 h at room temperature, followed by PBS washes. Visualization was performed using a DAB kit (ZLI‐9018, ZSGB‐BIO) according to the manufacturer's instructions. The slides were coverslipped with Cytoseal 60 and imaged using a digital scanner (Leica HS6, Germany). The images were analyzed utilizing Image J software.

### 2.16 Immunofluorescence Staining

Paraffin‐embedded sections were dewaxed, rehydrated, and subjected to microwave‐based antigen retrieval. HMC3 cells cultured on coverslips in a 24‐well plate were fixed with 4% PFA for 20 min and permeabilized with 1× PBS + 0.3% Triton X‐100 for 15 min. For immunofluorescence analysis, brain tissue sections and cells were incubated with primary antibodies (listed in Table S3) overnight at 4°C, followed by fluorescently labeled secondary antibodies for 2 h at room temperature. Fluorescently labeled secondary antibodies included Donkey Anti‐Goat IgG H&L (Alexa Fluor 488) at a dilution of 1:1000 (Abcam), CoraLite488‐conjugated Goat Anti‐Rabbit IgG H&L, and CoraLite594‐conjugated Goat Anti‐Rabbit/mouse IgG H&L at a dilution of 1:200 (Proteintech, Wuhan, China). Subsequently, brain slices and cells were counterstained with 4′, 6‐diamidino‐2‐phenylindole (DAPI, Beyotime Biotech) for 5 min and mounted with Mowiol before observation under a fluorescence microscope (Ni‐U, Nikon, Japan).

### 2.17 Electron Microscopy

At 28 days after HI insult, mice were humanely euthanized and underwent transcardial perfusion with a solution of 4% PFA and 2.5% glutaraldehyde in PBS following an initial flush with PBS. The brains were then carefully dissected and trimmed to obtain 1‐2 mm^3^ sections containing the corpus callosum. These sections were subsequently fixed in the same solution at 4°C overnight. Following dehydration with acetone, the tissues were embedded in epoxy 812 and sectioned into ultrathin slices (60–80 nm) using a Leica ultrathin microtome (Weztlar, Germany). The sections were stained with uranyl acetate and lead citrate before being examined under a transmission electron microscope (Tokyo, Japan). G‐ratios were determined by calculating the ratio of the inner axonal diameter to the total outer diameter (d/D) of myelinated axons using Image J software.

### 2.18 Statistical Analysis and Reproducibility

All measurements were conducted with a minimum of three independent replicates obtained from distinct experiments. Statistical evaluations for each experiment are detailed in the corresponding figure legends. The data presented are expressed as the mean ± SD. Shapiro–Wilk test was used to analyze the normal distribution. Nonparametric tests were employed for data that did not follow a normal distribution. For experiments involving multiple groups, one‐way ANOVA with Tukey's post hoc test was applied. Student's t‐test was utilized for comparisons between two groups. Statistical significance was determined using GraphPad Prism 8.1 (GraphPad Software, Inc., CA, USA). A *p* value < 0.05 was considered statistically significant across all types of statistical analyses.

## 3 Results

### 3.1 Effects of PPAR‐γ on Persistent Neurological Deficits in a Neonatal Mouse Model of HI‐Induced WMI

In this study, we examined the effects of intraperitoneal delivery of either vehicle, RSGI, or GW9662 over a period of 28 days on the neurofunctional deficits in the neonatal WMI model. The OFT was employed to assess the impact of PPAR‐γ on anxiety‐like behaviors in the WMI mice induced by HI insult. The results depicted in Figure 1C,D revealed no significant differences in total travel distance and average speed among animals in the four experimental groups. Notably, compared with the Sham group, mice in the WMI group showed significantly less time spent and shorter distance traveled in the central area of the open field, indicative of anxiety‐like behaviors. And mice in the WMI + RSGI group conferred a better performance with increasing time spent and longer distance traveled in the central area of the open field compared with that in the WMI group. Conversely, administration of GW9662 led to poorer performance in the OFT than of mice in the WMI group (Figure 1C,E).

To investigate the potential of PPAR‐γ activation by RSGI in preserving recognition memory in neonatal mice with WMI, NOR tests were conducted (Figure 1F). In the NOR testing period, mice from the WMI + RSGI group spent more time exploring the new object than mice from the WMI group, suggesting the preservation of recognition memory by RSGI treatment. Conversely, mice in the WMI + GW group showed reduced exploration time toward the novel object and a significant decrease in DI and RI compared to the WMI mice (Figure 1G–I), indicating an exacerbation of cognitive impairment.

Next, a beam‐walking test was conducted to assess motor coordination in mice following the exposure to HI injury. The results depicted in Figure 1J demonstrated that HI‐induced WMI mice exhibited prolonged traversal time and increased frequency of foot slippage on an 80‐cm long beam as compared to the Sham mice. Treatment with RSGI significantly improved the performance in beam‐walking test than that of mice in the WMI group, while mice from the WMI + GW group displayed exacerbated performance with longer transmit time and increasing frequency of foot slippage relative to the WMI group (Figure 1J).

Collectively, these findings suggest that PPAR‐γ activation facilitates neurofunctional recovery, as evidenced by the alleviation in anxiety‐like behaviors, enhancement of motor coordination, and mitigation of cognitive impairments induced by neonatal WMI. Conversely, while blockade of PPAR‐γ aggravated poor performances in these neurobehavior tests.

### 3.2 Effects of PPAR‐γ on Blood–Brain Barrier (BBB) Disruption and OLs Differentiation in the Neonatal WMI Model

**FIGURE 2:**
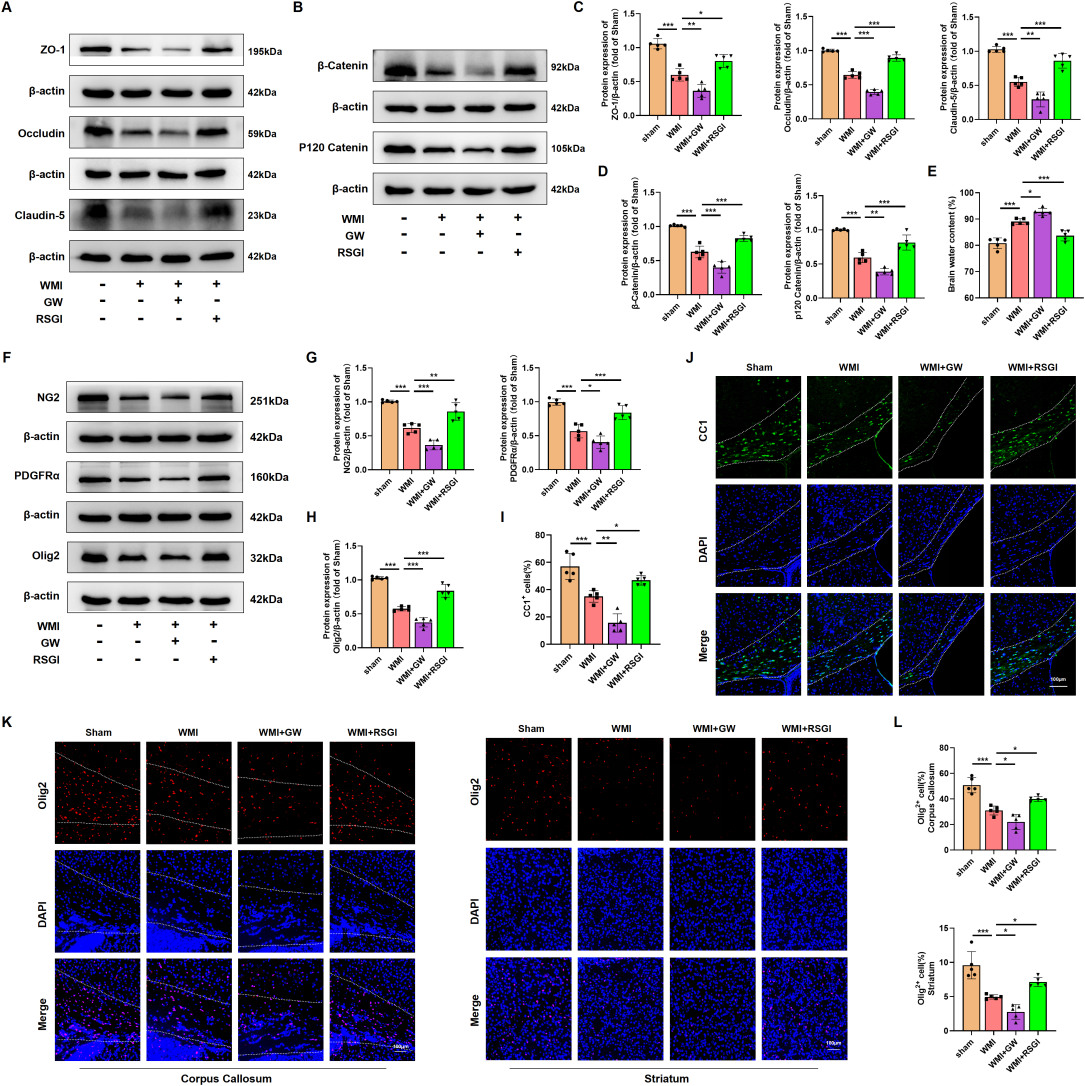
Effects of PPAR‐γ on BBB disruption and OLs differentiation in the neonatal WMI model. (A, B) Western blots depicting the expression of tight junction proteins and adherens junction proteins in WM 24 h post‐HI injury. The molecular weight marker (in kDa) is shown on the right. (C, D) Quantification of the western blots illustrated in (A, B). (E) The ratio of wet and dry in each group. (F) Western blots showing the levels of Olig2, PDGFRα, and NG2 in WM 72 h post‐HI injury. The molecular weight marker (in kDa) is indicated on the right. (G, H) Quantification of the western blots presented in (F). (I) Quantification of the percentage of CC1 positive cells as shown in (J). (J) Representative immunofluorescence images showing CC1 (green) and DAPI (blue) in the corpus callosum (outlined in white) at 28 days after HI injury. Scale bar, 100 μm. (K) Representative immunofluorescence images showing Olig2 (red) and DAPI (blue) in the corpus callosum (outlined in white) and striatum at 72 h after HI injury. Scale bar, 100 μm. (L) Quantification of the percentage of Olig2 positive cells as shown in (K). Data are expressed as fold induction over the sham. Graph displays mean ± SD values (*n* = 5 brains per condition). *p < 0.05, **p < 0.01, and ***p < 0.001, significance based on one‐way ANOVA with Tukey's post hoc test.

To determine the effects of PPAR‐γ on BBB disruption caused by HI in neonatal mice, brain water content and western blot were performed 24 h after WMI model establishment. The results of western blot illustrated in Figure 2A–D demonstrate that HI significantly induced the downregulation of tight junction proteins (ZO‐1, Occludin, and Claudin‐5) and adherens junction proteins (β‐Catenin and P120 Catenin), but this effect was effectively mitigated by treatment with RSGI. Notably, compared with the WMI group, GW9662 treatment further downregulated the expression levels of these proteins. The quantitative results of brain water content showed that RSGI treatment significantly ameliorated brain edema induced by HI insult, but edema extent was further aggravated after GW9662 treatment (Figure 2E). Overall, these findings suggest that PPAR‐γ protects BBB integrity against HI insult in a neonatal mouse model of WMI.

To study the effects of PPAR‐γ on OLs differentiation after neonatal HI, we performed western blot and immunofluorescence staining. Western blot analysis demonstrated downregulated levels of platelet‐derived growth factor receptor alpha (PDGFRα, an OL progenitor marker), neuron glia antigen‐2 (NG2, oligodendrocyte precursor cells (OPCs) marker), and oligodendrocyte transcription factor 2 (Olig2) 72 h after HI insult in the WMI group compared with the Sham group, while activation of PPAR‐γ with RSGI significantly increased the levels of these proteins (Figure 2F–H). Consistently, immunofluorescence staining further showed that HI resulted in obvious reduction in the density of Olig2‐positive cells in the corpus callosum and striatum compared with the Sham group, while RSGI treatment dramatically restored the density of Olig2‐positive cells (Figure 2K,L). Next, we conducted immunofluorescent staining for CC1 (a mature OL marker). Quantification of CC1‐positive cells in the corpus callosum 28 days post‐modeling showed a reduction in cell density following HI, while the forced activation of PPAR‐γ using RSGI was sufficient to upregulate the density of CC1‐positive cells (Figure 2I,J). Notably, GW9662 further exacerbated the reduction in protein levels of PDGFRα, Olig2, and NG2 and the density of Olig2‐positive or CC1‐positive cells at indicated time when compared with those in the WMI group (Figure 2). Overall, these findings demonstrate the protective impact of PPAR‐γ in mitigating OLs loss and promoting OLs differentiation in a neonatal mouse model of HI‐induced WMI.

### 3.3 Effects of PPAR‐γ on OLs Maturation, Myelination, and Synaptic Deficits in Neonatal WMI Mice Induced by HI

**FIGURE 3:**
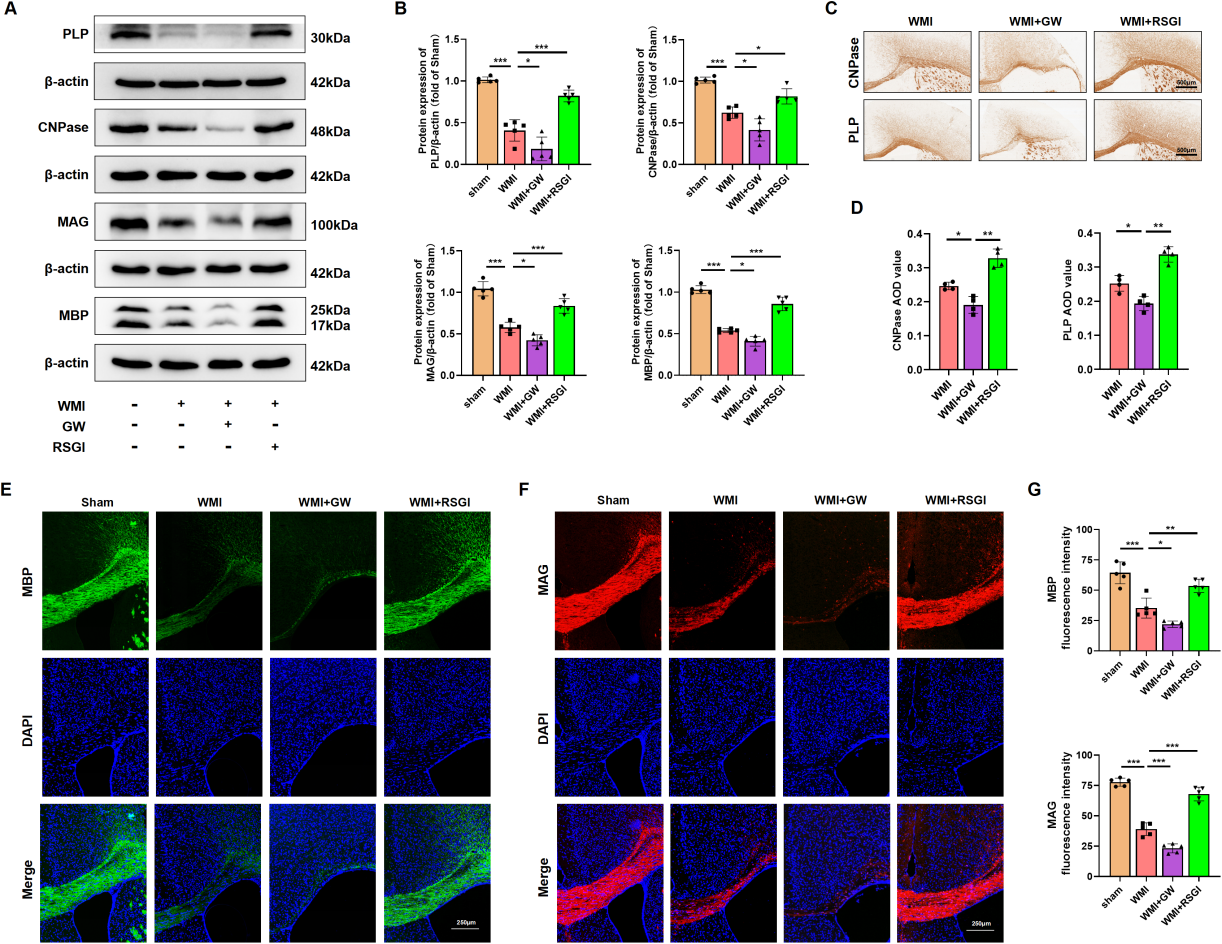
Effects of PPAR‐γ on OLs maturation in the neonatal WMI model. (A) Representative western blots for myelin proteins PLP, CNPase, MAG, and MBP in WM at 28 days after HI injury. Molecular weight marker (in kDa) is indicated on the right. (B) Quantification of western blots depicted in (A), presented as fold induction relative to the sham group. (C) Representative immunohistochemistry images displaying CNPase and PLP at 28 days after HI injury. Scale bar, 500 μm. (D) Statistical evaluation of immunohistochemistry staining for CNPase and PLP. (E, F) Representative immunofluorescence images of MBP (green), MAG (red), and DAPI (blue) in the corpus callosum at 28 days after HI injury. Scale bar, 250 μm. (G) Quantitative analysis of fluorescence intensity for MBP and MAG. Graph displays mean ± SD values (*n* = 4–5 brains per condition). *p < 0.05, **p < 0.01, and ***p < 0.001, significance based on one‐way ANOVA with Tukey's post hoc test.

**FIGURE 4:**
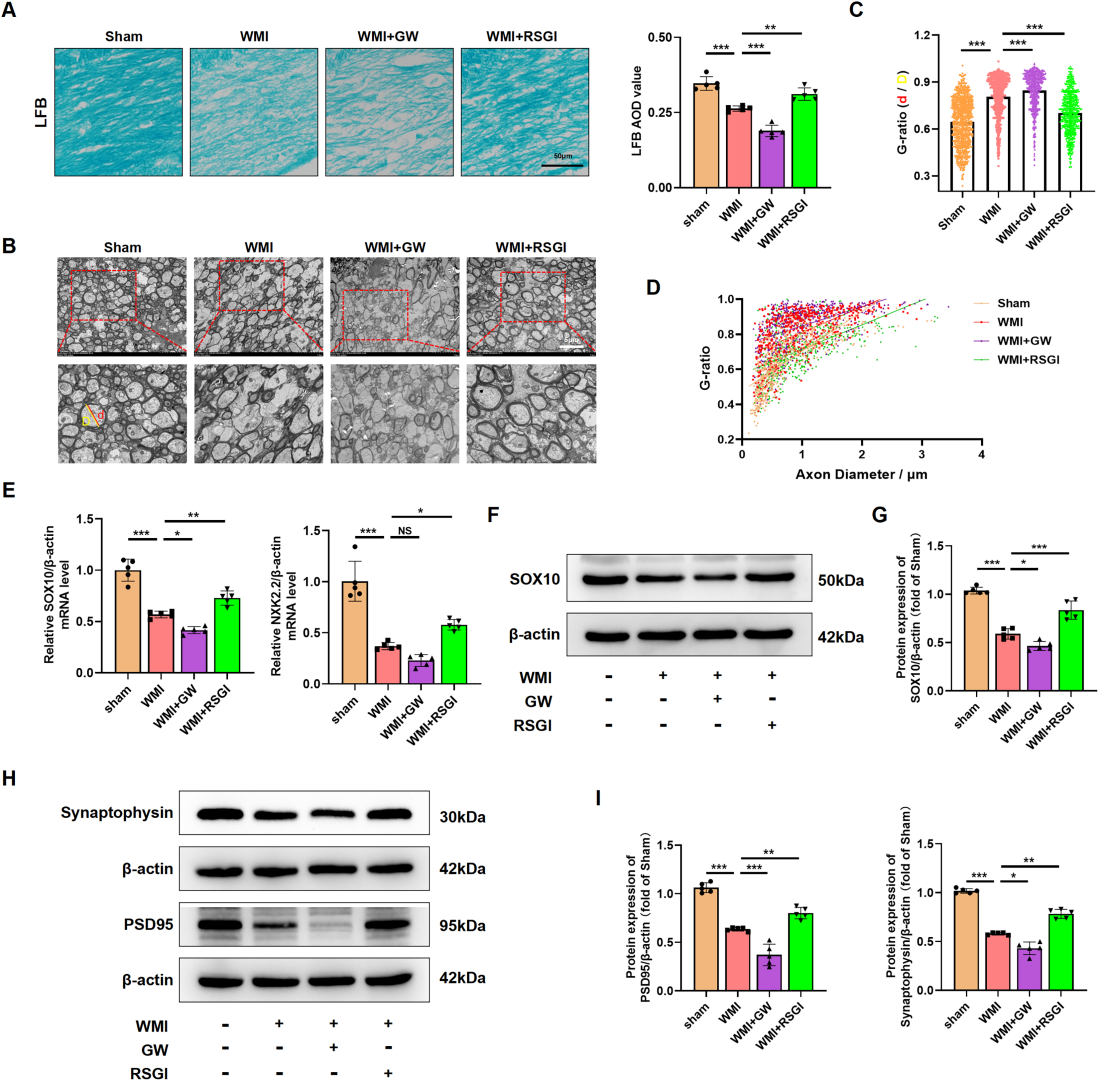
Effects of PPAR‐γ on myelin loss and synaptic deficits in the neonatal WMI model. (A) Representative images and quantitative analysis of LFB staining in WM at 28 days after HI injury. Scale bar, 500 μm. (B) Transmission electron micrographs revealing the ultrastructural characteristics of the corpus callosum at 28 days post‐HI insult. Scale bar, 5 μm. (C, D) Quantitative analysis of G‐ratios, with scatterplot depicting individual axonal g‐ratios relative to axonal diameters. *n* ≥ 100 axons counted per group, 4 mice per group. Statistical significance denoted by ***p < 0.001, determined by one‐way ANOVA followed by Tukey's post hoc test. Simple linear regression analysis of slopes in the scatterplot. (E) Representative qRT‐PCR analysis of SOX10 and NKX2.2 expression levels in WM at 28 days after HI injury, normalized to β‐actin levels. (F, G) Representative images and quantification of western blot analysis for SOX10 in WM at 28 day after HI injury. Molecular weight marker (in kDa) is indicated on the right. Results expressed as fold induction compared to the sham group. (H, I) Representative images and quantification of western blots for Synaptophysin and PSD95 in WM at 28 days after HI injury. Molecular weight marker (in kDa) is indicated on the right. Data are expressed as fold induction over the sham group. Graph represents mean ± SD values (*n* = 5 brains per condition). *p < 0.05, **p < 0.01, and ***p < 0.001, significance based on one‐way ANOVA with Tukey's post hoc test.

Given the protective effect of PPAR‐γ on OLs differentiation, we proceeded to investigate whether PPAR‐γ activation could expedite OLs maturation, promote remyelination, and improved synaptic deficits in a mouse model of neoantal WMI. We initially performed immunostaining assay and western blot analysis to determine the expression level of myelin proteins expressed in mature OLs 28 days after model establishment, respectively. As expected, western blot analysis revealed that the expression levels of Proteolipid Protein (PLP), 2′, 3′‐cyclic‐nucleotide 3′‐phosphodiesterase (CNPase), myelin‐associated glycoprotein (MAG), and myelin basic protein (MBP) were greatly diminished in the WMI group compared with the Sham group, while RSGI treatment demonstrated higher levels of these proteins than those of the WMI group (Figure 3A,B). Furthermore, immunostaining results exhibited a similar tendency as compared with western blot analysis of the same marker (Figure 3C–G). LFB staining revealed an improvement in the arrangement of nerve fibers and an increase in the average optical density (AOD) values in the WMI mice following RSGI treatment (Figure 4A). Similar to the results of LFB staining, analyses of G‐ratio from transmission electronic microscopy (TEM) revealed significantly fewer myelinated axons in the corpus callosum of HI brains compared to the Sham animals, while this trend was substantially mitigated by RSGI treatment (Figure 4B–D).

To assess the impact of PPAR‐γ activation on transcriptional activity in OLs and subsequent enhancement of OLs differentiation, qRT‐PCR analysis was performed to quantify the mRNA levels of differentiation‐associated transcriptional factors including homeobox gene Nkx2.2 and the SRY‐related HMG‐box gene SOX10 at 28 days after modeling. As depicted in Figure 4E, we found that PPAR‐γ activation with RSGI treatment significantly upregulated the mRNA levels of both Nkx2.2 and SOX10 in HI‐induced mice, suggesting a promotion of OL transcription. Additionally, western blots analysis showed RSGI treatment substantially reversed the HI‐induced downregulation in the protein level of SOX10, thereby providing further validation for the role of PPAR‐γ activation in promoting transcriptional activity in OLs (Figure 4F,G).

Next, western blot was utilized to assess protein levels of the presynaptic protein (synaptophysin) and postsynaptic protein (PSD‐95), both crucial for synapse formation and remodeling. The results indicated decreased protein levels of synaptophysin and PSD‐95 in the WMI group as compared to the Sham group, suggesting synaptic deficits. However, this downregulation greatly was prevented by RSGI treatment (Figure 4H,I). In contrast, GW9662 treatment exacerbated impaired myelination and deteriorated synaptic damage as evidenced by reduced levels of myelin proteins and transcription factors of OLs, further decrease in AOD values from LFB staining, increased G‐ratio, and decreased levels of synaptic protein (Figure 4).

Taken together, these results indicate that PPAR‐γ activation promotes OLs maturation, myelination, and attenuates synaptic deficits in neonatal WMI mice induced by HI.

### 3.4 Effects of PPAR‐γ on the Activation of Astrocyte and Microglial Polarization Induced by HI in Neonatal Mice

**FIGURE 5:**
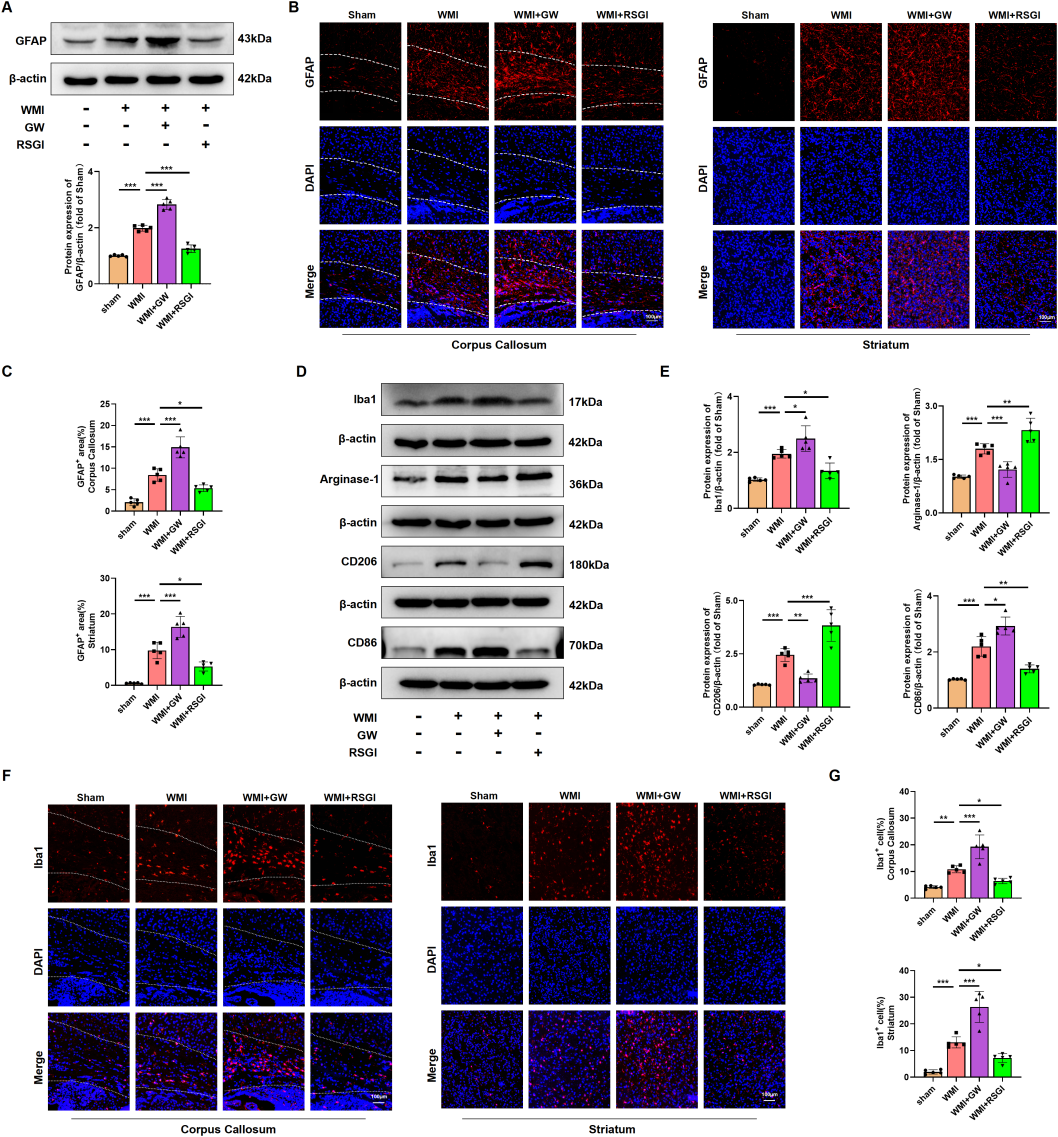
Effects of PPAR‐γ on the activation of microglia and astrocyte in the neonatal WMI model. (A) Representative images and quantitative analysis of western blot for GFAP in WM at 24 h after HI injury. Molecular weight marker (in kDa) is indicated on the right. Data are expressed as fold induction over the sham group. (B) Representative immunofluorescence images depicting GFAP (red), and DAPI (blue) in the corpus callosum (outlined in white) and striatum at 24 h after HI injury. Scale bar, 100 μm. (C) Quantification of GFAP‐positive area shown in (B). (D) Representative images of western blots for Iba1, CD86, CD206, and Arginase‐1 in WM at 24 h after HI injury. Molecular weight marker (in kDa) is indicated on the right. (E) Quantification of western blot results displayed in (D). Data are expressed as fold induction over the sham. (F) Representative immunofluorescence images illustrating Iba1 (red), and DAPI (blue) in the corpus callosum (outlined in white) and striatum at 24 h after HI injury. Scale bar, 100 μm. (G) Quantitative analysis of the percentage of Iba‐1‐positive cells shown in (F). Graph displays mean ± SD values (*n* = 5 brains per condition). *p < 0.05, **p < 0.01, and ***p < 0.001, significance based on one‐way ANOVA with Tukey's post hoc test.

The involvement of activation of microglia and astrocyte is widely acknowledged for its substantial contribution to the regulation of the neuroinflammation, which is crucial in the pathogenesis of WMI in premature infants [11]. To validate an inhibitory effect of PPAR‐γ on the activation of astrocyte and microglia, western blot was initially conducted in the WMI mice 24 h after HI injury. Notably, results showed that protein levels of Iba1 (a classical bio‐marker of microglia activation) and GFAP (a classical bio‐marker of reactive astrogliosis) were upregulated in the WMI group compared with the Sham group. RSGI treatment reversed this effect in HI‐induced mice (Figure 5A,D,E). Moreover, GFAP‐positive cells (Figure 5B,C) and Iba1‐positive cells (Figure 5F,G) in each group as detected by immunofluorescent staining showed the same expression trend as the protein expression of GFAP and Iba1. However, inhibiting PPAR‐γ using GW9662 upregulated the protein levels of Iba1 and GFAP and increased the density of both Iba1‐positive cells and GFAP‐positive cells, suggesting a heightened activation of microglia and astrocyte.

A microglial transition from M2 phenotype to M1 phenotype plays a pivotal role in the microglial activation and the subsequent neuroinflammatory response [11, 33]. To investigate the effect of PPAR‐γ on microglial polarization, we performed western blot to detect protein levels of M1‐specific markers (CD86) and M2‐specific markers (CD206, Arginase‐1). Upregulated levels of both M1‐specific and M2‐specific markers were observed in the WMI group; however, RSGI treatment reduced levels of M1‐specific markers, while remarkably upregulating those of M2‐specific markers, whereas GW9662 treatment increased the levels of M1‐specific markers and decreased M2‐specific markers in HI‐induced mice, indicating microglial polarization toward the M1 phenotype (Figure 5D,E).

Overall, these data indicate that PPAR‐γ activation exhibits an inhibitory effect on activation of microglia and astrocyte and promotes microglial polarization to the M2 phenotype in the neonatal HI WMI model.

### 3.5 HMGB1/NF‐κB Pathway Is Involved in the Amelioration of Neuroinflammatory Response After Activation of PPAR‐γ in the Neonatal WMI Model

**FIGURE 6:**
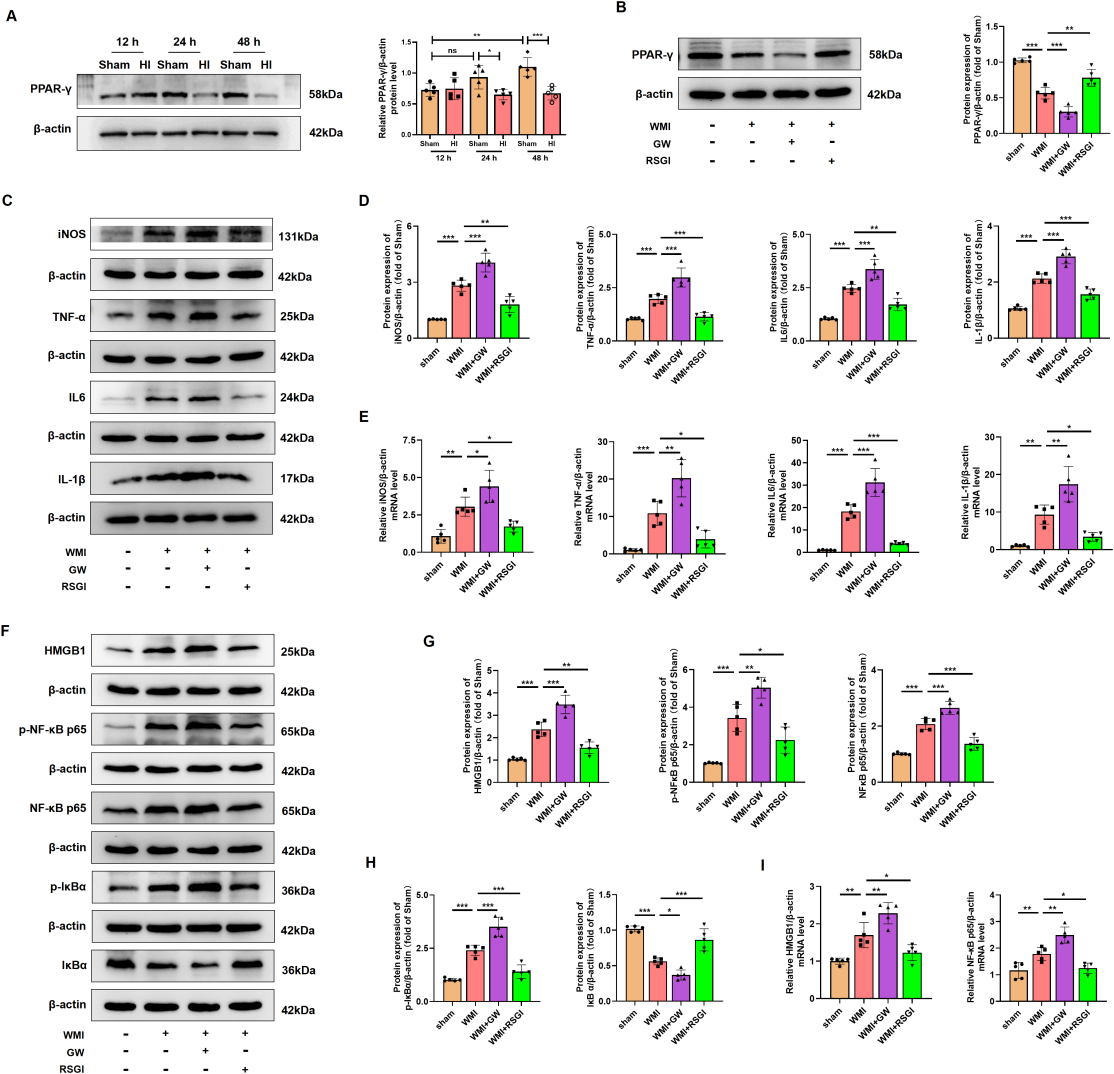
Effects of PPAR‐γ on neuroinflammation in the neonatal WMI model. (A) Representative images and quantitation of western blots for PPAR‐γ at indicated time after HI injury. (B) Representative images and quantitative analysis of western blots for PPAR‐γ in different groups at 24 h after HI injury. Molecular weight marker (in kDa) is indicated on the right. Data are expressed as fold induction over the sham. (C) Representative images of western blots for iNOS, TNF‐α, IL6, and IL‐1β in WM at 24 h after HI injury. Molecular weight marker (in kDa) is indicated on the right. (D) Quantification of western blots shown in (C), expressed as fold induction over sham. (E) Representative qRT‐PCR analysis of iNOS, TNF‐α, IL6, and IL‐1β expression levels in WM at 24 h after HI injury, normalized to β‐actin. (F) Representative images of western blots for HMGB1, p‐NF‐κB p65, NF‐κB p65, p‐IκBα, and IκBα in WM at 24 h after HI injury, with molecular weight markers (kDa) on the right. (G, H) Quantification of western blots shown in (F), expressed as fold induction over sham. (I) Representative qRT‐PCR analysis of HMGB1 and NF‐κB p65 expression levels in WM at 24 h after HI injury, normalized to β‐actin. Graph displays mean ± SD values (*n* = 5 brains per condition). *p < 0.05, **p < 0.01, and ***p < 0.001, significance based on one‐way ANOVA with Tukey's post hoc test.

To determine the protein expression of PPAR‐γ in the neonatal WMI model, western blot was performed at 12 h, 24 h, and 48 h after HI injury. HI led to a reduction in protein expression of PPAR‐γ at 24 h and 48 h after HI injury (Figure 6A). Notably, the decreased level of PPAR‐γ protein induced by HI insult was reversed by treatment with RSGI. Conversely, treatment with GW9662 exacerbated the downregulation of PPAR‐γ protein levels (Figure 6B). We further elucidate the regulatory function of PPAR‐γ in neuroinflammatory response through western blot and qRT‐PCR of inflammatory cytokines. The WMI group exhibited significantly higher levels of pro‐inflammatory cytokines (iNOS, TNF‐α, IL‐1β, and IL6) at both protein (Figure 6C,D) and mRNA levels (Figure 6E) compared with the sham group, while RSGI treatment decreased HI‐induced expression of these cytokines. Conversely, blocking PPAR‐γ with GW9662 significantly worsened inflammatory response in HI‐induced mice as revealed by the upregulated expression levels of pro‐inflammatory cytokines (Figure 6C–E).

The translocation and release of High Mobility Group Protein 1 (HMGB1) play important roles in inflammatory response in neonatal HI brain injury [34]. Assessing cytosol protein via western blot showed that HI injury increased the cytosol transfer of HMGB1 at 24 h after HI. Treatment with RSGI led to a reduction in cytosol HMGB1 levels (Figure 6F,G). Activation of the NF‐κB pathway is associated with HMGB1 expression and the post‐WMI neuroinflammation [35, 36]. To further investigate the specific mechanisms of PPAR‐γ‐mediated alleviation of inflammation under WMI condition, NF‐κB activation was assessed at 24 h after HI in neonatal mice. The results showed an upregulation in the protein levels of p‐NF‐κB p65, NF‐κB p65, and p‐IκBα and a downregulation in the protein levels of IκBα in the WMI mice, indicating activation of NF‐κB pathway (Figure 6F–H). Moreover, qRT‐PCR analysis (Figure 6I) mirrored the protein expression trends of HMGB1 and NF‐κB p65. However, GW9662 treatment remarkably exacerbated the activation of HMGB1/NF‐κB pathway as revealed by elevated levels of cytosol HMGB1, p‐NF‐κB p65, p‐IκBα, and NF‐κB p65 proteins, and lower protein levels of IκBα (Figure 6F–I).

Therefore, based on the above evidence, we speculate that PPAR‐γ activation might exert inhibitory effects on the excessive neuroinflammation in the WMI mice via suppressing the HMGB1/NF‐κB pathway.

### 3.6 NRF‐2/KEAP1 Pathway Is Involved in the Amelioration of Excessive Oxidative Stress After Activation of PPAR‐γ in the Neonatal WMI Model

**FIGURE 7:**
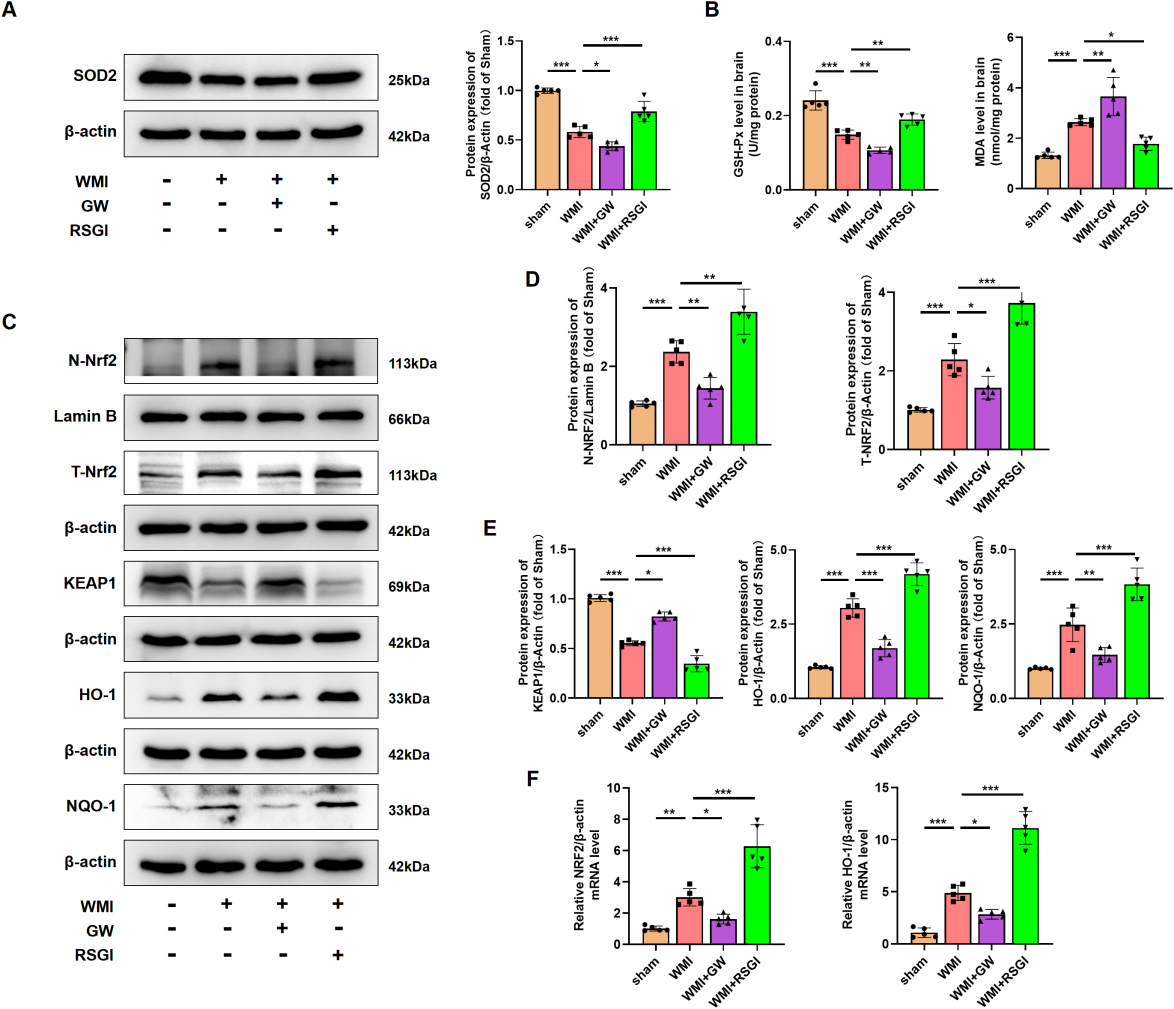
Effects of PPAR‐γ on excessive oxidative stress in the neonatal WMI model. (A) Representative images and quantification of western blots for SOD2 in WM at 24 h after HI injury. Molecular weight marker (in kDa) is indicated on the right. Data are expressed as fold induction over the sham group. (B)Measurement of MDA and GSH‐Px levels in WM at 24 h after HI injury. (C) Representative images of western blots for T‐NRF2, N‐NRF2, KEAP1, HO‐1, and NQO‐1 in WM at 24 h after HI injury. Molecular weight marker (in kDa) is indicated on the right. (D, E) Quantification of western blots for T‐NRF2, N‐NRF2 (D), KEAP1, HO‐1, and NQO‐1 (E). Data are expressed as fold induction over the sham. (F) Representative qRT‐PCR analysis of NRF2 and HO‐1 expression levels in WM at 24 h after HI injury. Expression levels normalized to β‐actin. Graph displays mean ± SD values (*n* = 5 brains per condition). *p < 0.05, **p < 0.01, and ***p < 0.001, significance based on one‐way ANOVA with Tukey's post hoc test.

In addition to the excessive neuroinflammatory response, microglial activation also induced excessive oxidative stress in cerebral WMI. Given that activation of PPAR‐γ with RSGI inhibited the microglial activation and subsequent inflammation, we further investigated the inhibitory effects and mechanism of PPAR‐γ on the oxidative stress induced by HI in neonatal mice. We initially quantified the levels of MDA, antioxidant activity (superoxide dismutase (SOD)2 and GSH‐Px), and found that HI induced higher levels of MDA and lower levels of GSH‐Px and SOD2 as compared with the Sham group, while RSGI treatment remarkably downregulated the levels of MDA with concomitant upregulation in the levels of GSH‐Px and SOD2 as compared with the WMI group (Figure 7A,B).

NRF2 is a major regulator against oxidative stress. Several scientific studies have demonstrated the therapeutic potential of targeting the NRF2/Kelch‐like ECH‐associated protein 1 (KEAP1) pathway in HI‐induced brain injury [37, 38, 39]. Western blot was further utilized to detect NRF2/KEAP1 pathway, and analyses showed that RSGI treatment significantly increased the expression levels of T‐NRF2, heme oxygenase‐1 (HO‐1), NAD(P)H dehydrogenase quinone 1 (NQO‐1), and decreased the expression levels of KEAP1 and facilitated the nuclear translocation of NRF2 as compared with the Sham group (Figure 7C–E). qRT‐PCR analysis showed upregulation in mRNA levels of NRF2 and HO‐1 in the RSGI‐treated WMI group compared with the WMI group (Figure 7F), confirming the antioxidant effects of PPAR‐γ activation against HI‐induced WMI. However, GW9662 treatment significantly suppressed the activation of NRF2, increased expression levels of KEAP1, downregulated levels of antioxidant proteins (HO‐1, NQO‐1, SOD2, and GSH‐Px), and elevated the levels of MDA compared with the WMI group (Figure 7C–F).

Therefore, based on the above evidence, we speculate that PPAR‐γ activation might exert inhibitory effects on the excessive oxidative stress in WMI mice via activating the NRF2/KEAP1 pathway.

### 3.7 HMGB1/NF‐κB Pathway Is Involved in the Regulation of Microglial Polarization After Activation of PPAR‐γ in HMC3 Cells After OGD

**FIGURE 8:**
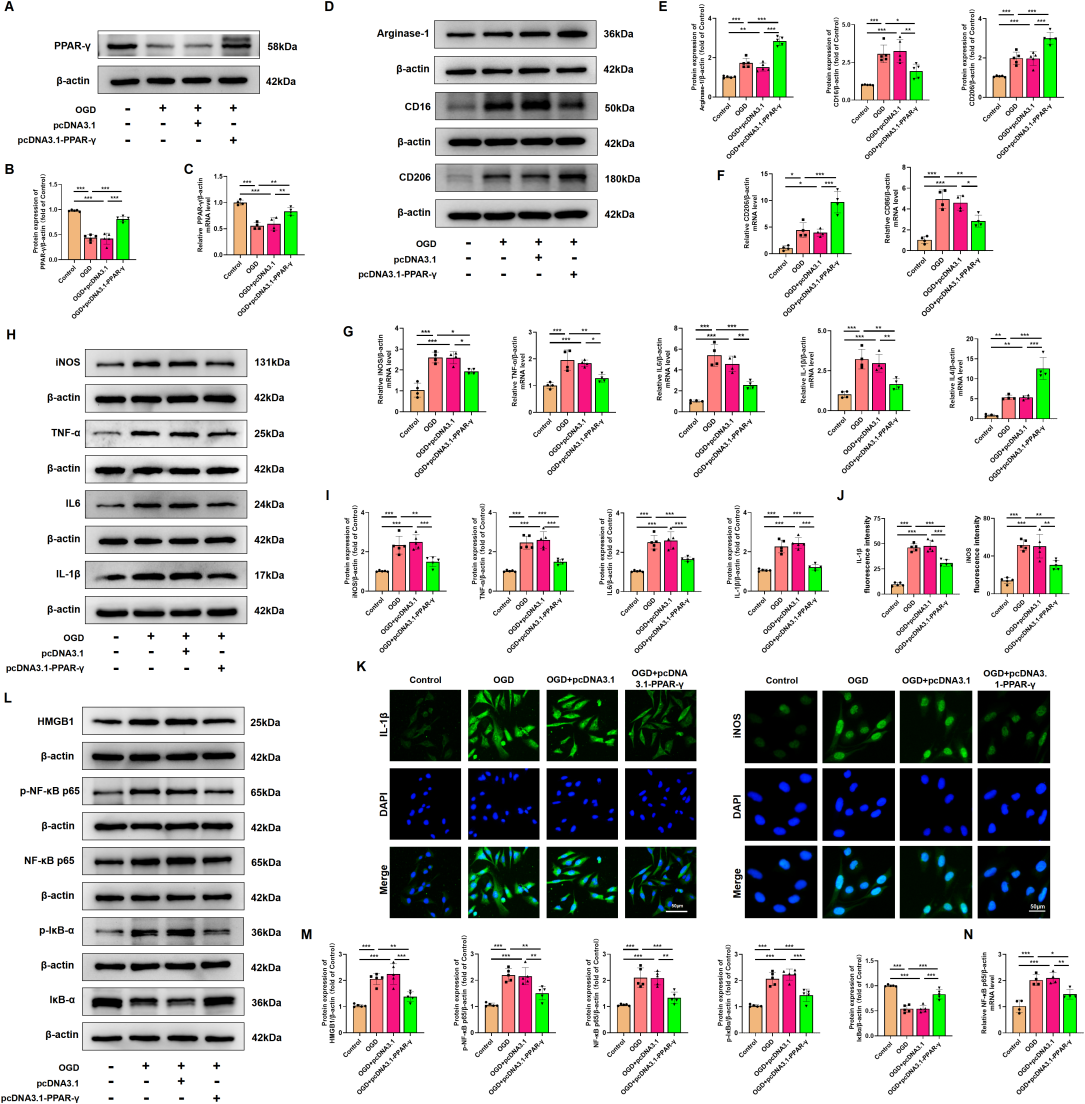
Overexpressing PPAR‐γ shifts microglial polarization from M1 to M2 phenotype and alleviates inflammatory response in HMC3 cells after OGD. (A, B) Representative images and quantification of western blots for PPAR‐γ in HMC3 cells. Molecular weight marker (in kDa) is indicated on the right. Data are expressed as fold induction over the control. (C) Representative qRT‐PCR analysis of PPAR‐γin HMC3 cells. (D, E) Representative images and quantification of western blots for Arginase‐1, CD16, and CD206 in HMC3 cells. Data are expressed as fold induction over the control. (F, G) Representative qRT‐PCR analysis of CD206, CD86, TNF‐α, IL‐1β, iNOS, IL6, and IL4 expression levels in HMC3 cells. (H, I) Representative images and quantification of western blots for iNOS, TNF‐α, IL6, and IL‐1β in HMC3 cells. Data are expressed as fold induction over the control. (J) Statistical analysis of fluorescence intensity of IL‐1β and iNOS. (K) Representative immunofluorescence images displaying iNOS (green), IL‐1β (green) and DAPI (blue) in HMC3 cells. Scale bar, 50 μm. (L, M) Representative images and quantification of western blots for HMGB1, p‐NF‐κB p65, NF‐κB p65, p‐IκBα, and IκBα in HMC3 cells. Data are expressed as fold induction over the control. (N) Representative qRT‐PCR analysis of NF‐κB p65 expression levels in HMC3 cells. mRNA levels were normalized against those of β‐actin. Histograms show mean ± SD values (*n* = 4–5 cultures per condition). *p < 0.05, **p < 0.01, and ***p < 0.001, significance based on one‐way ANOVA with Tukey's post hoc test.

**FIGURE 9:**
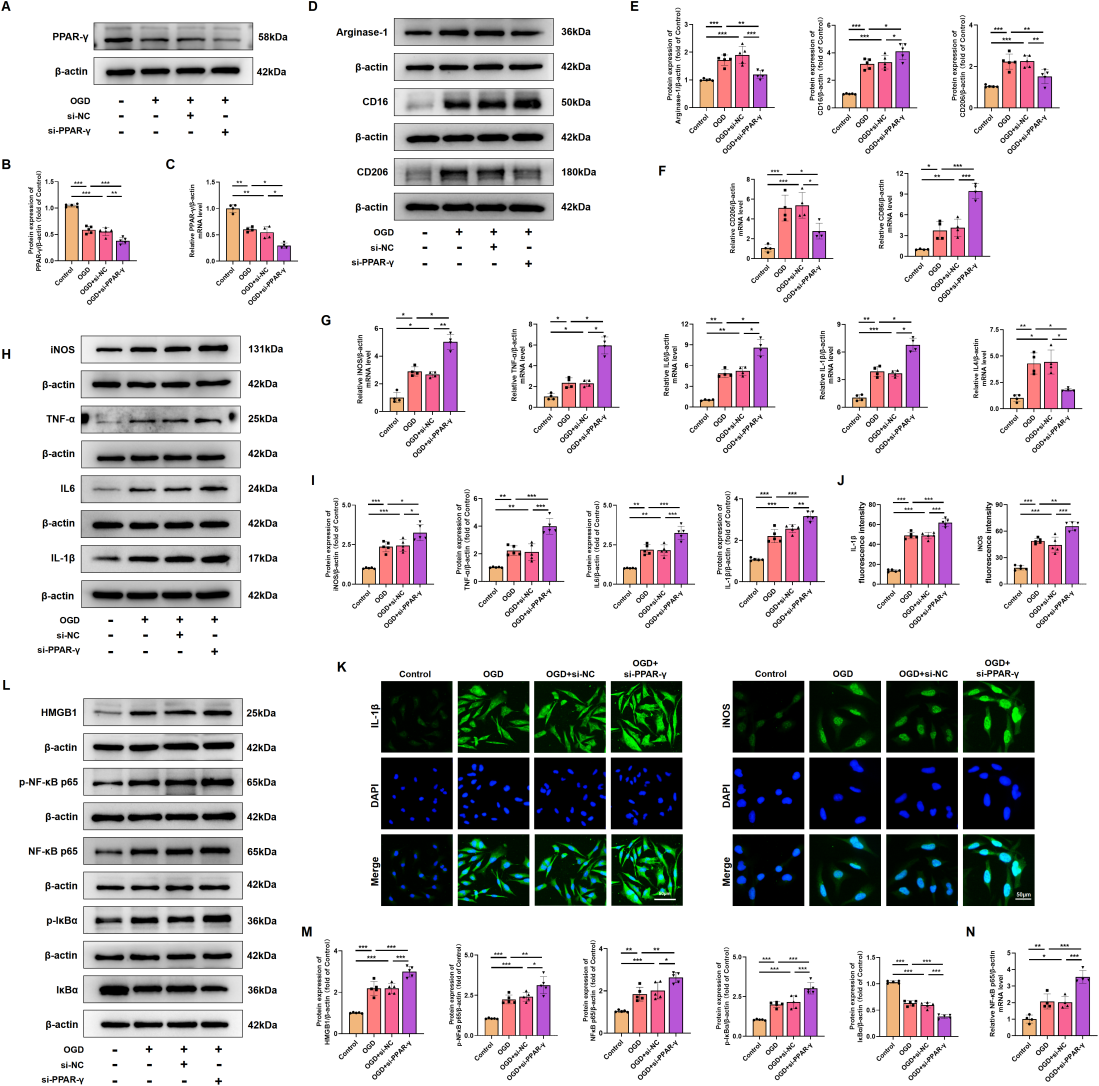
Silencing PPAR‐γ aggravates M1/M2‐polarization state of microglia and inflammatory response in HMC3 cells after OGD. (A, B) Representative images and quantification of western blots for PPAR‐γ in HMC3 cells. Molecular weight marker (in kDa) is indicated on the right. Data are expressed as fold induction over the control. (C) Representative qRT‐PCR analysis of PPAR‐γ in HMC3 cells. Levels were normalized against those of β‐actin. (D, E) Representative images and quantification of western blots for Arginase‐1, CD16, and CD206 in HMC3 cells. Data are expressed as fold induction over the control. (F, G) Representative qRT‐PCR analysis of CD206, CD86, TNF‐α, IL‐1β, iNOS, IL6, and IL4 expression levels in HMC3 cells. (H, I) Representative images and quantification of western blots for iNOS, TNF‐α, IL6, and IL‐1β in HMC3 cells. Data are expressed as fold induction over the control. (J) Statistical analysis of fluorescence intensity of IL‐1β and iNOS. (K) Representative immunofluorescence images displaying iNOS (green), IL‐1β (green) and DAPI (blue) in HMC3 cells. Scale bar, 50 μm. (L, M) Representative images and quantification of western blots for HMGB1, p‐NF‐κB p65, NF‐κB p65, p‐IκBα, and IκBα in HMC3 cells. Data are expressed as fold induction over the control. (N) Representative qRT‐PCR analysis of NF‐κB p65 expression levels in HMC3 cells. mRNA levels were normalized against those of β‐actin. Histograms show mean ± SD values (*n* = 4–5 cultures per condition). *p < 0.05, **p < 0.01, and ***p < 0.001, significance based on one‐way ANOVA with Tukey's post hoc test.

Considering the constraints in systemic administration layout, it is imperative to ascertain the role of PPAR‐γ in microglia through further advancements. OGD was performed to simulate HI conditions, and the effect of OGD on HMC3 cell viability in different time periods was examined through the CCK‐8 assay. Results showed that the viability of cells exposed to OGD for 18 h was reduced to 60.17% ± 3.01% (Figure S2A); thus, OGD for 18 h was chosen in subsequent experiments. Subsequent in vitro experiments revealed that the protein expression level of PPAR‐γ was downregulated in HMC3 cells exposure to OGD injury (Figure S2B). Furthermore, utilizing small interfering RNA and plasmid techniques, we successfully manipulated PPAR‐γ expression levels and confirmed the transfection efficiency. The map and sequencing information of recombinant expression vector of pcDNA 3.1‐PPAR‐γ, and sequencing information of si‐PPAR‐γ were shown in Figure S1. Results from qRT‐PCR and western blot showed that the administration of pcDNA 3.1‐PPAR‐γ effectively increased PPAR‐γ expression at both mRNA and protein levels (Figure S2C,D), while si‐PPAR‐γ led to its downregulation in HMC3 cells (Figure S2E,F). In OGD‐treated HMC3 cells, the decrease in the mRNA and protein levels of PPAR‐γ was significantly counteracted upon PPAR‐γ overexpressing (Figure 8A–C), but was aggravated upon knockdown of PPAR‐γ (Figure 9A–C).

Consistent with in vivo findings, western blot analysis showed that overexpressing PPAR‐γ significantly decreased the protein expression levels of M1‐specific markers (CD16), and increased the protein expression levels of M2‐specific markers (CD206, Arginase‐1) in HMC3 following OGD injury (Figure 8D,E). However, silencing PPAR‐γ showed higher expression levels of M1‐specific markers, and lower expression levels of M2‐specific markers compared with that in the OGD‐preconditioned HMC3 cells (Figure 9D,E). qRT‐PCR analysis of CD86 and CD206 further confirmed the effect of PPAR‐γ activation on microglial transition from M1 to M2 phenotype (Figures 8F, 9F). Moreover, we assessed inflammatory response in OGD‐preconditioned HMC3 cells. Compared with the OGD‐treated HMC3 cells, overexpressing PPAR‐γ obviously downregulated pro‐inflammatory cytokines, such as iNOS, TNF‐α, IL‐6, and IL‐1β both at protein and mRNA levels (Figure 8G–I), whereas silencing PPAR‐γ showed higher expression of pro‐inflammatory cytokines induced by OGD injury both at protein and mRNA levels than that in OGD‐treated HMC3 cells (Figure 9G–I). Quantitative immunofluorescence showed a similar trend in the expression of iNOS and IL‐1β in each group as observed in western blot analysis (Figures 8J,K and 9J,K).

Based on the aforementioned data, we evaluated the effects of PPAR‐γ on HMGB1/NF‐κB pathway. Compared with the control group, OGD induced cytosol transfer of HMGB1, promoted the phosphorylation of NF‐κB p65 and IκBα, upregulated the protein levels of NF‐κB p65, and downregulated the protein level of IκBα. However, overexpressing PPAR‐γ reversed this effect (Figure 8L,M). qRT‐PCR analysis showed that mRNA levels of NF‐κB p65 were decreased in PPAR‐γ‐overexpressing HMC3 cells after OGD injury (Figure 8N). Notably, knockdown of PPAR‐γ elevated expression levels of cytosol HMGB1, p‐NF‐κB p65, NF‐κB p65, p‐IκBα, and elevated mRNA levels of NF‐κB p65, as well as decreased expression levels of IκBα compared with OGD‐treated HMC3 cells (Figure 9L–N). Thus, our findings suggest that the involvement of PPAR‐γ is pivotal in modulating the HMGB1/NF‐κB pathway and inflammatory response in HMC3 cells after OGD.

### 3.8 NRF‐2/KEAP1 Pathway Is Involved in the Reduction in Excessive Oxidative Stress After Overexpression of PPAR‐γ in HMC3 Cells After OGD

**FIGURE 10:**
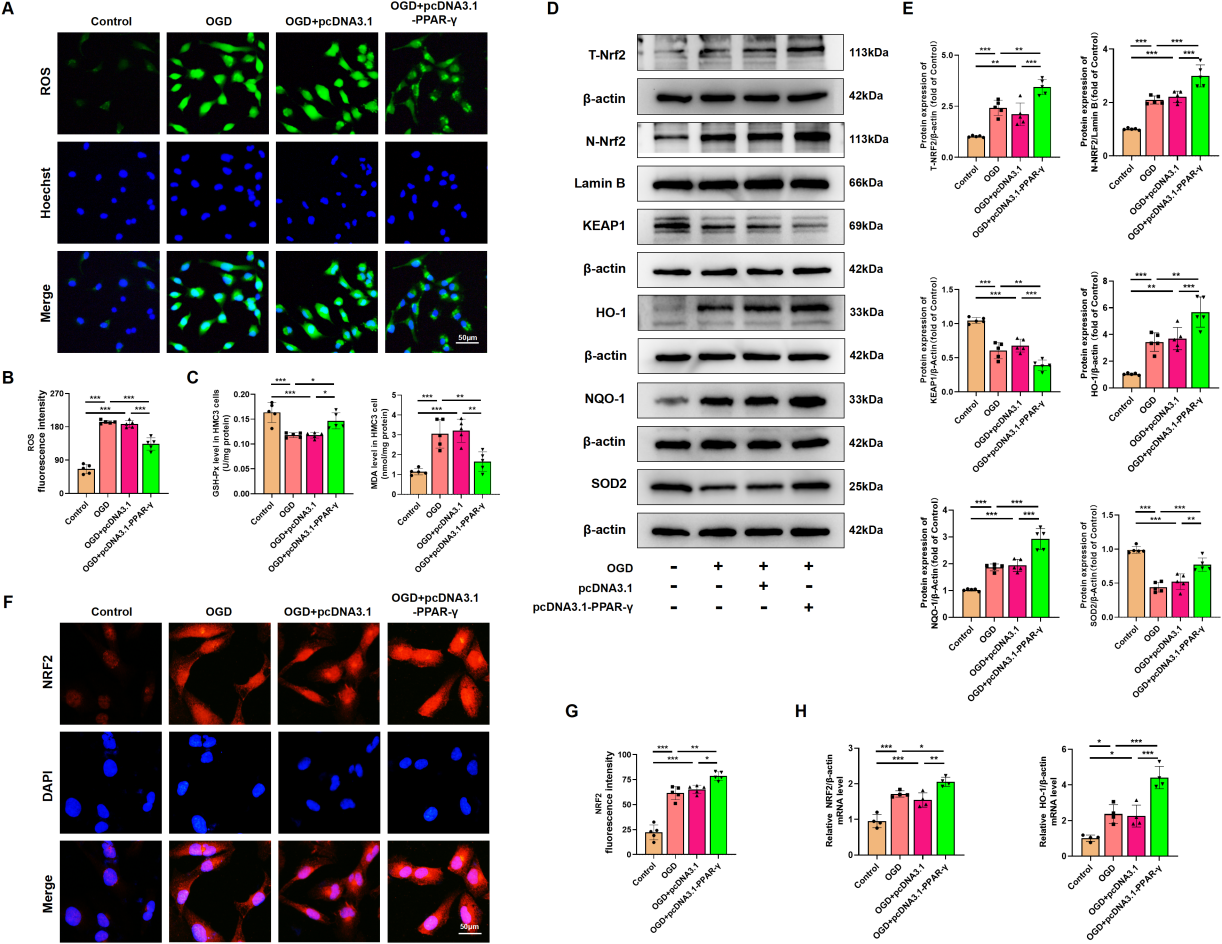
Overexpressing PPAR‐γ mitigates excessive oxidative stress in HMC3 cells after OGD. (A) Representative immunofluorescence images displaying ROS (green) and Hoechst (blue) in HMC3 cells. Scale bar, 50 μm. (B) Statistical analysis of fluorescence intensity of ROS. (C) Measurement of MDA and GSH‐Px levels in HMC3 cells after OGD injury. (D) Representative images of western blots for T‐NRF2, N‐NRF2, KEAP1, HO‐1, NQO‐1, and SOD2 in HMC3. Molecular weight marker (in kDa) is indicated on the right. (E) Quantification of the western blot results in (D) was performed, and data are presented as fold induction relative to the control. (F) Representative immunofluorescence displaying NRF2 (red) and DAPI (blue) in HMC3 cells. Scale bar, 50 μm. (G) Statistical analysis of fluorescence intensity of NRF2. (H) qRT‐PCR analysis revealed the expression levels of NRF2 and HO‐1 in HMC3 cells, normalized to β‐actin. Histograms show mean ± SD values (*n* = 4–5 cultures per condition). *p < 0.05, **p < 0.01, and ***p < 0.001, significance based on one‐way ANOVA with Tukey's post hoc test.

**FIGURE 11:**
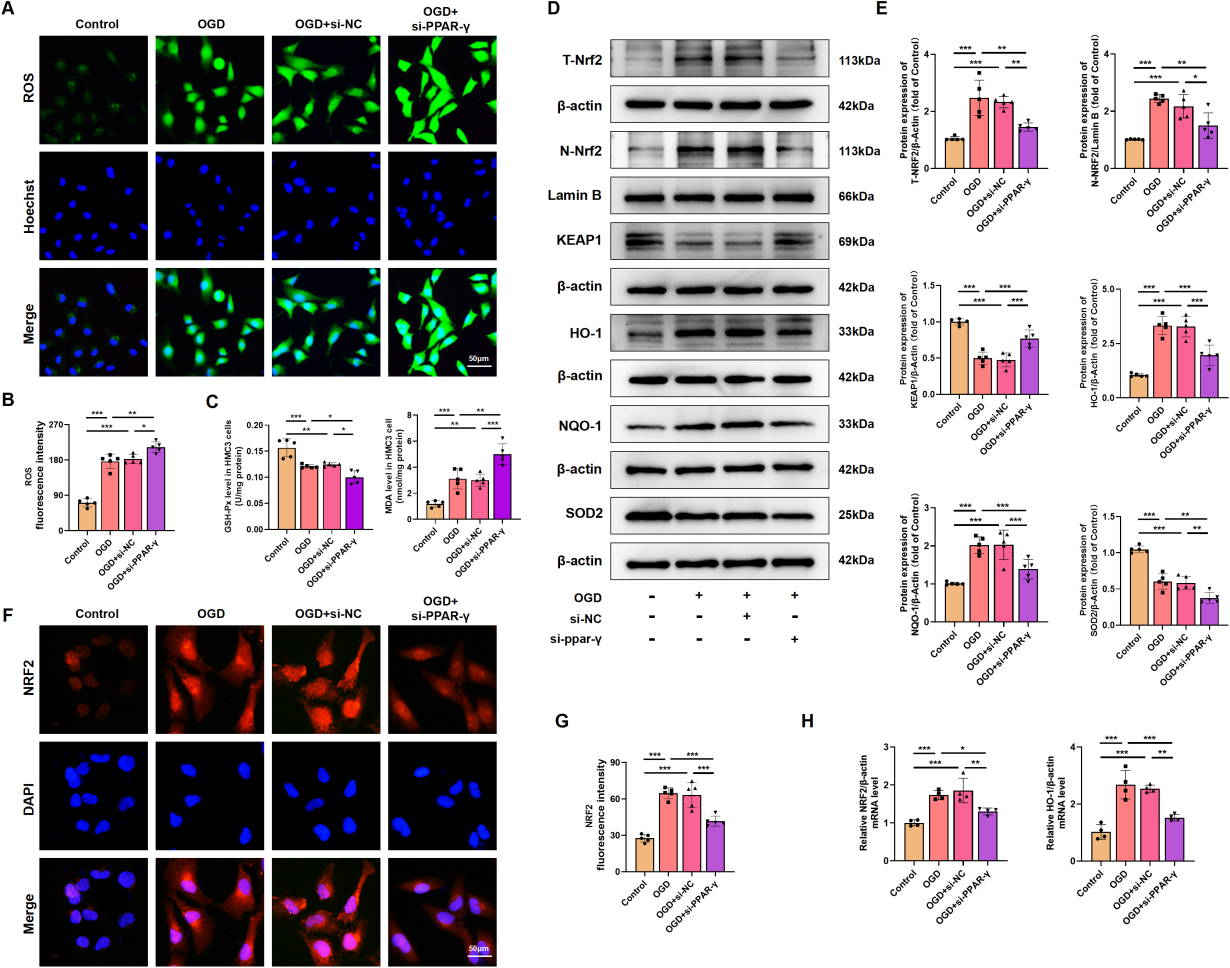
Silencing PPAR‐γ exacerbates oxidative stress in HMC3 cells after OGD. (A) Representative immunofluorescence images displaying ROS (green) and Hoechst (blue) in HMC3 cells. Scale bar, 50 μm. (B) Statistical analysis of fluorescence intensity of ROS. (C) Measurement of MDA and GSH‐Px levels in HMC3 cells after OGD injury. (D) Representative images of western blots for T‐NRF2, N‐NRF2, KEAP1, HO‐1, NQO‐1, and SOD2 in HMC3. Molecular weight marker (in kDa) is indicated on the right. (E) Quantification of the western blot results in (D) was performed, and data are presented as fold induction relative to the control. (F) Representative immunofluorescence displaying NRF2 (red) and DAPI (blue) in HMC3 cells. Scale bar, 50 μm. (G) Statistical analysis of fluorescence intensity of NRF2. (H) qRT‐PCR analysis revealed the expression levels of NRF2 and HO‐1 in HMC3 cells, normalized to β‐actin. Histograms show mean ± SD values (*n* = 4–5 cultures per condition). *p < 0.05, **p < 0.01, and ***p < 0.001, significance based on one‐way ANOVA with Tukey's post hoc test.

Given that activation of PPAR‐γ attenuated the microglial activation both in vivo and in vitro, we further evaluated the inhibitory effects and mechanism of PPAR‐γ on the oxidative stress induced by OGD in HMC3 cells. To measure intracellular ROS produced by oxidative stress, the ROS analysis kit was used in OGD‐treated HMC3 cells. Our results indicated that ROS fluorescence intensity in OGD‐treated HMC3 cells was reduced upon administration of pcDNA 3.1‐PPAR‐γ (Figure 10A,B), while it was elevated with si‐PPAR‐γ administration (Figure 11A,B). These findings suggest that PPAR‐γ activation effectively prevents the upregulation of ROS produced by oxidative stress in OGD‐treated HMC3 cells, while inhibition of PPAR‐γ fails to scavenge ROS. Further investigation into the molecular mechanism of PPAR‐γ revealed increased expression levels of total NRF2 (T‐NRF2), nuclear NRF2 (N‐NRF2), and elevated amounts of antioxidant proteins including HO‐1, NQO‐1, SOD2, and GSH‐Px, as well as decreased levels of KEAP1 and MDA in PPAR‐γ‐overexpressing HMC3 cells after OGD injury (Figure 10C–E). However, silencing PPAR‐γ decreased the expression of T‐NRF2, N‐NRF2, antioxidant proteins (HO‐1, NQO‐1, SOD2, and GSH‐Px), and increased the levels of KEAP1 and MDA (Figure 11C–E) in OGD‐treated HMC3 cells. Consistent trends were observed in fluorescence intensity (Figures 10F,G and 11F,G) and mRNA levels of NRF2 and HO‐1 compared to western blot analysis (Figures 10H and 11H).

Overall, our findings suggest that overexpression of PPAR‐γ activates the NRF‐2/KEAP1 antioxidant pathway, while knockdown of PPAR‐γ inhibits this pathway in OGD‐treated HMC3 cells.

## 4 Discussion

WMI is the most prevalent type of the injuries affecting the developing preterm brain, with existing treatment options being limited [5]. Survivors of preterm birth with WMI often experience long‐term neurodevelopmental disabilities [2, 40]. The aim of this study is to identify new and effective therapeutic targets to enhance myelination and promote recovery in premature infants with WMI.

PPAR‐γ has been extensively investigated for its role in modulating glucose and lipid metabolism, energy homeostasis, and functions as a therapeutic target for the treatment of metabolic disorders, for instance diabetes mellitus [41, 42]. Recently, the neuroprotective effects of PPAR‐γ in various central nervous system (CNS) disease models have garnered significant attention. Various studies have demonstrated that activation of PPAR‐γ presents a promising therapeutic target for the management of traumatic brain injury (TBI) [20], ischemic brain injury [19], Parkinson's disease [43], and Alzheimer's disease [44] in animal models. This is achieved by reducing neuronal injury, inhibiting oxidative stress and promoting redox homeostasis, suppressing inflammatory responses, enhancing cell proliferation and neurogenesis, and other neuroprotective mechanisms [24]. Moreover, emerging evidence suggests that PPAR‐γ also confers beneficial effects on WMI following TBI [45], cerebral ischemia [46], and spinal cord injury [47]. A study by Yao et al. demonstrated that alpha‐asaronol promotes the proliferation of OPCs and ameliorates dysmyelination in neonatal rats after HI insult by upregulating PPAR‐γ [28], highlighting the protective role of PPAR‐γ in OLs differentiation and myelination in HI‐induced WMI. However, beyond its effects on OLs maturation, the protective mechanisms of PPAR‐γ against other pathological changes and neurodevelopmental disorders in preterm infants following WMI remain poorly understood.

The maturation of the OL lineage occurs in four sequential stages: the OL progenitor, the pre‐OL (also referred to as late OL progenitor), the immature OL, and the mature OL, which finally synthesizes myelin. In our study, we developed a neonatal male murine model of WMI at P5, which coincides with a developmental time window analogous to the human gestational age of 23–32 weeks, characterized by a peak in immature non‐myelinating OLs [27]. Moreover, OGD‐treated HMC3 cells were used to simulate microglia exposed to HI in vivo, along with in vivo experiments assessing the potential therapeutic effects and mechanism of PPAR‐γ in neonatal HI‐induced WMI.

Preterm survivors with WMI commonly exhibit neurodevelopmental disorders, including behavioral issues, motor impairments, and cognitive deficits [8, 40]. Our study corroborated previous findings by showing impaired recognition memory in our animal model using NOR tests, with WMI mice displaying a marked decline in discriminative capacity. Significantly, we discovered that activating PPAR‐γ with RSGI enhanced recognition memory in WMI mice, leading to substantial improvements in the DI. Conversely, inhibiting PPAR‐γ with GW9662 further decreased discriminative ability in WMI mice. Additionally, results from the OFT and beam‐walking test revealed that PPAR‐γ activation with RSGI resulted in increased time spent and distance traveled in the center of the open field, as well as reduced transit time and foot slippage frequency in the beam‐walking test, indicating that PPAR‐γ activation may ameliorate anxiety and motor impairments in WMI. Conversely, blocking PPAR‐γ with GW9662 exacerbated anxiety and motor impairments induced by HI.

The reduction in OLs and disturbances in OLs maturation during brain development have been linked to motor and cognitive deficits in juvenile individuals with a history of neonatal WMI [48]. In this study, consistent with prior research, HI injury resulted in OLs depletion and reduced brain myelination, characterized by decreased expression of OPCs markers (PDGFRα and NG2), reduced density of Olig2‐positive cells and mature OLs (CC1‐positive cells), diminished levels of myelination‐associated proteins (PLP, CNPase, MAG, and MBP), decreased expression of transcriptional factors (Olig2, Nkx2.2, and SOX10), and decreased myelin density and thickness as observed through electron microscopy and LFB staining. Notably, activation of PPAR‐γ with RSGI mitigated OLs loss, enhanced myelination, and increased the number of myelinated axons and myelin thickness in WMI mice. Conversely, inhibition of PPAR‐γ with GW9662 exacerbated HI‐induced OLs depletion and impaired myelination.

The diverse long‐term sequelae observed in preterm birth with WMI cannot be solely attributed to straightforward myelin sheath dysplasia. Notably, in the growing studies, synaptic damage is thought to strongly correlate with this diversity. Synapse formation and remodeling plays a vital role in the development of immature brain [49]. Xiong et al. have documented synaptic damage in a rat model of HI brain injury, leading to cognitive and motor impairments [50]. Studies have indicated that PPAR‐γ influences synaptic plasticity, with PPAR‐γ signaling pathway being integral to fostering synapse formation, mitigating synaptic dysfunction, and thus preventing neurological deficits [51, 52]. Our initial assessment of synapse formation in HI‐induced WMI revealed a reduction in synaptic density, aligning with prior research findings. Furthermore, our investigation confirmed the impact of PPAR‐γ on synaptic deficits post‐HI, demonstrating that PPAR‐γ activation with RSGI mitigated the decline in synaptic protein expression (synaptophysin and PSD‐95) linked to synaptic transmission, synaptogenesis, and synaptic plasticity. Conversely, inhibition of PPAR‐γ with GW9662 exacerbated HI‐induced synaptic loss in WMI. While alterations in synaptic proteins were observed, direct evidence of changes in synaptic function remains inconclusive. Further studies using electrophysiological methods are needed to validate our findings and provide a more comprehensive understanding of synaptic activity.

In a prior study involving a rat model of WMI, it was demonstrated that the preservation of BBB integrity is crucial for promoting myelination [53]. Multiple lines of evidence indicate that activation of PPAR‐γ can safeguard the BBB from damage, leading to improved outcomes in various CNS diseases such as stroke [29], TBI [20], and chronic cerebral hypoxia [54]. Consistent with previous research, our findings revealed that HI disrupted the BBB, characterized by reduced expression of tight junction and adherens junction proteins. Activation of PPAR‐γ with rosiglitazone prevented BBB disruption and ameliorated brain edema, whereas inhibition of PPAR‐γ with GW9662 exacerbated BBB breakdown.

The neuroprotective effects of PPAR‐γ on HI‐induced WMI in neonatal mice have been demonstrated in detail, while the underlying mechanisms of PPAR‐γ are not yet fully elucidated. In preterm birth, HI insult triggers a series of signaling cascades that induce inflammation, generate ROS and oxidative stress, and cause glutamate‐evoked excitotoxic injury in the brain WM lesion, thereby impeding OL differentiation and maturation [6, 11]. Zhang et al. reported an increased secretion of pro‐inflammatory cytokines in the cerebrospinal fluid of neonates following HI injury; these elevations correlate with poor neurological prognosis and may strongly correlate with the occurrence of cerebral palsy [55]. Further analysis revealed that the inflammatory response of microglial cells in the cerebral white matter can generate free radicals and trigger excessive oxidative stress [56]. Several studies have suggested that inhibiting inflammatory responses and reducing oxidative stress could be potential targets for the treatment of neonatal WMI [6, 11]. Oxidative stress and inflammation are interlinked consequences of microglial cell reactions to HI‐induced WMI. Microglia are observed preferentially within the developing white matter and essential for normal brain development [33]. Under physiological condition, Microglia are typically maintained in a relatively inactive state. However, in response to hypoxia, ischemia, and inflammation, microglial cells can become activated and polarized toward a pro‐inflammatory M1 phenotype (expressing CD16/32 and CD86), leading to the production of various pro‐inflammatory mediators (such as iNOS, TNF‐α, IL‐1β, and IL‐6), chemokines, ROS, and reactive nitrogen species, which collectively result in oxidative and inflammatory injury. In contrast, polarization of microglia toward an M2 phenotype (expressing CD206 and Arginase‐1) results in the secretion of anti‐inflammatory mediators (such as TGF‐β, IL‐4, and IL‐10), antioxidant enzymes (such as HO‐1, NQO‐1, SOD, and GSH‐Px), and nutritional factors that promote OL differentiation and myelination [15, 16, 17]. Therefore, following experiments in this study should aim to improve microglia modulation and promote myelination following neonatal HI injury.

Numerous studies utilizing PPAR‐γ activators or agonists have demonstrated a pronounced decrease in the secretion of pro‐inflammatory mediators and an increase in the secretion of anti‐inflammatory cytokines, along with an upregulation in the production of antioxidant defense enzymes [24, 57]. These effects of PPAR‐γ enhancement through its activators or agonists have ultimately resulted in the amelioration of inflammatory responses and a reduction in pro‐oxidative reactions. The correlation between PPAR‐γ activation and its anti‐inflammatory and antioxidant responses in various CNS disorders has been extensively elucidated [30, 58]. Nonetheless, the impact of PPAR‐γ on microglia‐mediated neuroinflammation and oxidative stress in a murine model of WMI, as well as the underlying mechanisms, remain incompletely understood. Our study aimed to investigate the effects of PPAR‐γ on neuroinflammation and oxidative stress induced by activated microglia both in vivo and in vitro.

The current findings corroborate this observation, with the WMI model demonstrating increased expression of M1‐ and M2‐specific markers upon model establishment. Activation of PPAR‐γ using RSGI suppressed the M1 phenotype in microglia, reduced the production of pro‐inflammatory cytokines and ROS, and enhanced the M2 phenotype, fostering the generation of anti‐inflammatory cytokines and antioxidant enzymes. Conversely, inhibition of PPAR‐γ with GW9662 exacerbated microglial polarization toward the M1 phenotype, decreased the presence of M2 microglia, and elevated the release of pro‐inflammatory mediators and ROS, leading to inflammatory cascades and heightened oxidative stress. In our in vitro experiments, we utilized small interfering RNA and plasmid techniques to modulate PPAR‐γ expression in HMC3 cells. Consistent with our in vivo results, overexpression of PPAR‐γ using pcDNA 3.1‐PPAR‐γ effectively dampened microglial activation, promoted polarization toward the M2 phenotype, thereby mitigating inflammatory responses and oxidative stress. Conversely, blocking PPAR‐γ with si‐PPAR‐γ exacerbated inflammatory responses and oxidative stress by promoting M1 polarization and microglial activation in OGD‐treated HMC3 cells. Therefore, the exploration of the underlying molecular mechanisms of PPAR‐γ predominantly focuses on microglial activation and polarization, ensuing inflammatory reactions, and oxidative stress.

As an endogenous damage‐associated molecular pattern (DAMP), HMGB1 plays a crucial role in mediating inflammation and the innate immune response in various neurological diseases including neonatal HI brain injury [34, 59, 60]. In the inflammatory conditions, HMGB1 can be released passively from damaged or necrotic cells and actively secreted by microglia. Extracellularly released HMGB1 binds to different cell surface receptors, such as pattern recognition receptors (PRRs). This ultimately leads to the phosphorylation of NF‐κB (IκB) and its translocation into the nucleus, results in imbalanced microglial polarization, and initiating gene transcription and the expression of inflammatory mediators [35, 36]. Notably, numerous studies have suggested that targeting the HMGB1/NF‐κB signaling pathway could be potential therapeutic strategy for treating HI‐induced brain injury in neonates [59, 61]. Previous research has demonstrated that PPAR‐γ can inhibit HMGB1/NF‐κB signaling pathway and then suppresses inflammatory responses in CNS diseases [62, 63]. Consistent with previous studies, our in vivo experiments showed that HI insult induced an increase in the expression, cytosol translocation, and extracellular release of HMGB1 along with upregulation in the phosphorylation of NF‐κB p65 and IκBα as well as IκBα degradation, all indicating activation of the HMGB1/NF‐κB signaling pathway. However, activation of the HMGB1/NF‐κB was reversed by activation of PPAR‐γ using RSGI. Conversely, GW9662‐induced inhibition of PPAR‐γ exacerbated cytosol translocation and secretion of HMGB1 while leading to stronger phosphorylation of NF‐κB p65 and IκBα and inhibited IκBα degradation, which suggesting more severe activation of the HMGB1/NF‐κB signaling pathway. In line with the in vivo experiments, our in vitro results showed that PPAR‐γ overexpression using pcDNA 3.1‐PPAR‐γ suppressed OGD‐induced activation of the HMGB1/NF‐κB signaling pathway, thereby promoting microglial polarization to M2 phenotype and mitigating inflammatory responses. On the contrary, PPAR‐γ blocking using si‐PPAR‐γ aggravated inflammatory responses via excessive activation of HMGB1/NF‐κB signaling pathway in OGD‐treated HMC3 cells. These findings indicate that PPAR‐γ suppresses HMGB1/NF‐κB signaling pathway and modulates microglial polarization, thereby mitigating inflammatory response. These findings provide compelling evidence supporting the modulation of microglial polarization and subsequent generation of pro‐inflammatory mediators by PPAR‐γ through the HMGB1/NF‐κB signaling pathway.

In addition to HMGB1/NF‐κB, PPAR‐γ also governs the prototypical redox‐sensing NRF2 [64], which is widely acknowledged as a transcription factor serving as the principal regulator of cellular antioxidant defense mechanisms [65]. Under normal circumstances, NRF2 is sequestered in the cytosol by KEAP1, subsequently ubiquitinated and degraded [66]. Upon exposure to various stressors such as hypoxia, ischemia, and inflammation, the cells generate ROS, leading to the liberation of NRF2 from KEAP1 and its translocation from the cytoplasm to the nucleus. Once in the nucleus, NRF2 binds to the antioxidant responsive element (ARE), thereby inducing antioxidative responses by promoting the transcription of downstream antioxidant and detoxification genes such as HO‐1, NQO‐1, GSH‐Px, SOD, and so on [66]. In earlier studies, Yang et al. reported that NRF2−/− mice showed aggravated cerebral damage, inflammation, and oxidative stress following neonatal HI injury [37]. Additionally, our previous investigations have highlighted the antioxidative properties of NRF2 activation in HI‐induced brain damage [38]. Recent in vitro studies have demonstrated that pharmaceutical agents targeting NRF2 can suppress pro‐inflammatory reactions and excessive oxidative stress in OGD‐treated microglia, while this effect was nullified when NRF2 expression was silenced using NRF2 siRNA [39]. Consequently, we postulated that NRF2 plays a significant role in microglial activation and subsequent oxidative stress, thereby facilitating pathological recovery and enhancing outcomes in WMI induced by HI events in premature infants. In accordance with previous studies, our study revealed that PPAR‐γ activation by RSGI reduced KEAP1 protein levels and increased T‐NRF2 and N‐NRF2 protein levels, and a downstream target HO‐1, NQO‐1, and SOD2 in HI‐induced brain WMI. Furthermore, our results demonstrated that PPAR‐γ activation by RSGI downregulated MDA levels, a marker of oxidative stress, and upregulated GSH‐Px levels, an essential endogenous antioxidant, in WM tissues of HI mice. On the contrary, GW9662‐induced inhibition of PPAR‐γ led to a reduction in both T‐NRF2 and N‐NRF2 protein levels, exacerbating oxidative stress as evidenced by elevated MDA levels and decreased GSH‐Px, HO‐1, NQO‐1, and SOD2 levels.

Consistent with the in vivo experiments, in vitro studies have demonstrated that overexpression of PPAR‐γ using pcDNA 3.1‐PPAR‐γ activates the NRF2 signaling pathway, resulting in microglial polarization to M2 phenotype and attenuation of oxidative stress in HMC3 cells after OGD injury. On the contrary, PPAR‐γ blocking using si‐PPAR‐γ aggravated oxidative stress in OGD‐treated HMC3 cells by further suppressing the NRF2 signaling pathway. These findings suggest involvement of PPAR‐γ in modulating microglial phenotype and subsequent oxidative stress via activation of NRF2 signaling pathway in HI‐induced WMI.

There are limitations in this experiment. Firstly, systemic drug administration may not provide a comprehensive understanding of the impacts and mechanisms of PPAR‐γ on WMI in preterm infants. Therefore, developing a microglia‐specific PPAR‐γ promoter through gene editing technology could be a promising approach for exploring the involvement of microglial PPAR‐γ in WMI. Furthermore, there is a lack of relevant research on the communication between microglia and OPCs or brain vascular endothelial cells in the in vitro studies. Strengthening our in vitro findings could be achieved by utilizing a transwell co‐culture system that involves primary microglia and OPCs or brain vascular endothelial cells exposed to OGD. Finally, in our study, there is a lack of relevant research regarding whether HMGB1 or NRF2 modulates the activation of PPAR‐γ in neonatal WMI. This will be elucidated in our future study by employing either an HMGB1 inhibitor or an NRF2 inhibitor.

## 5 Conclusion

In summary, we have elucidated the significant role of PPAR‐γ in the pathogenesis of HI‐induced WMI in neonatal mice. Our findings reveal that HMGB1/NF‐κB and NRF2 signaling pathways are involved in the regulation of microglial activation and polarization after PPAR‐γ activation. Consequently, PPAR‐γ attenuates inflammatory responses and oxidative stress, leading to enhanced OLs differentiation, myelination, and improving long‐term neurological outcomes in premature infants with WMI. These insights offer a new mechanistic understanding of WMI and propose that targeting PPAR‐γ may represent a promising therapeutic approach for WMI in preterm neonates.

## Author Contributions

M.F. and Z.L. designed the experiments. M.F. conducted the majority of the research and drafted the manuscript. Q.Y. and J.O. helped create the figures and write parts of the manuscript. M.F., J.L., and J.Z. analyzed the data. M.F., Z.L., and J.Z. contributed to critical revisions of the manuscript. All authors contributed to the article and approved the final manuscript.

## Ethics Statement

The animal experiments in this study were conducted in compliance with guidelines for the Care and Use of Laboratory Animals from the National Institute of Health and received approval from the Experimental Animal Ethics Committee at Wenzhou Medical University.

## Conflicts of Interest

The authors declare no conflicts of interest.

## Supporting information


Appendix S1.

## Data Availability

All the datasets supporting the conclusions of this article are available from the corresponding author on reasonable request if necessary.
