# Supplementary material for: The Neuroprotective Mechanisms of PPAR‐γ: Inhibition of Microglia‐Mediated Neuroinflammation and Oxidative Stress in a Neonatal Mouse Model of Hypoxic‐Ischemic White Matter Injury
Source: CNS Neurosci Ther. 2024 Nov 4;30(11):e70081. doi: 10.1111/cns.70081 (PMC11534457; doi:10.1111/cns.70081)
Supplement: Supplementary file 1 — Appendix S1. [file CNS-30-e70081-s001.doc]

**The neuroprotective mechanisms of PPAR-γ: Inhibition of microglia-mediated neuroinflammation and oxidative stress after hypoxic-ischemic white matter injury**

**Mingchu Fang^1,2,3,4^, Qianqian Yu^1,2^, Jiahao Ou^1,2^, Jia Lou^1,2^, Jianghu Zhu^1,2,3,4^ and Zhenlang Lin^1,2,3,4^,***

1 Department of Neonatology, The Second Affiliated Hospital and Yuying Children’s

Hospital of Wenzhou Medical University, Wenzhou, Zhejiang, China;

2 The Second School of Medicine, Wenzhou Medical University, Wenzhou, Zhejiang,

China;

3 Key Laboratory of Perinatal Medicine of Wenzhou, Wenzhou, Zhejiang, China;

4 Key Laboratory of Structural Malformations in Children of Zhejiang Province,

Wenzhou, Zhejiang, China

***Corresponding author: Zhenlang Lin, Email: linzhenlang@hotmail.com**

S1

**Supplementary Information**

**Figure S1.** The map and sequencing results of recombinant expression vector of pcDNA 3.1-PPAR-γ, and sequencing results of si-PPAR-γ………….......… ..S2-S4

**Figure S2.** PPAR-γ expression in HMC3 cells after different treatment…………… S5

**Table S1.** Primer information for mouse…………………………………….........…S6

**Table S2.** Primer information for human…………………………………….........…S7

**Table S3.** Primary Antibodies……………………………………....................…S8-S9


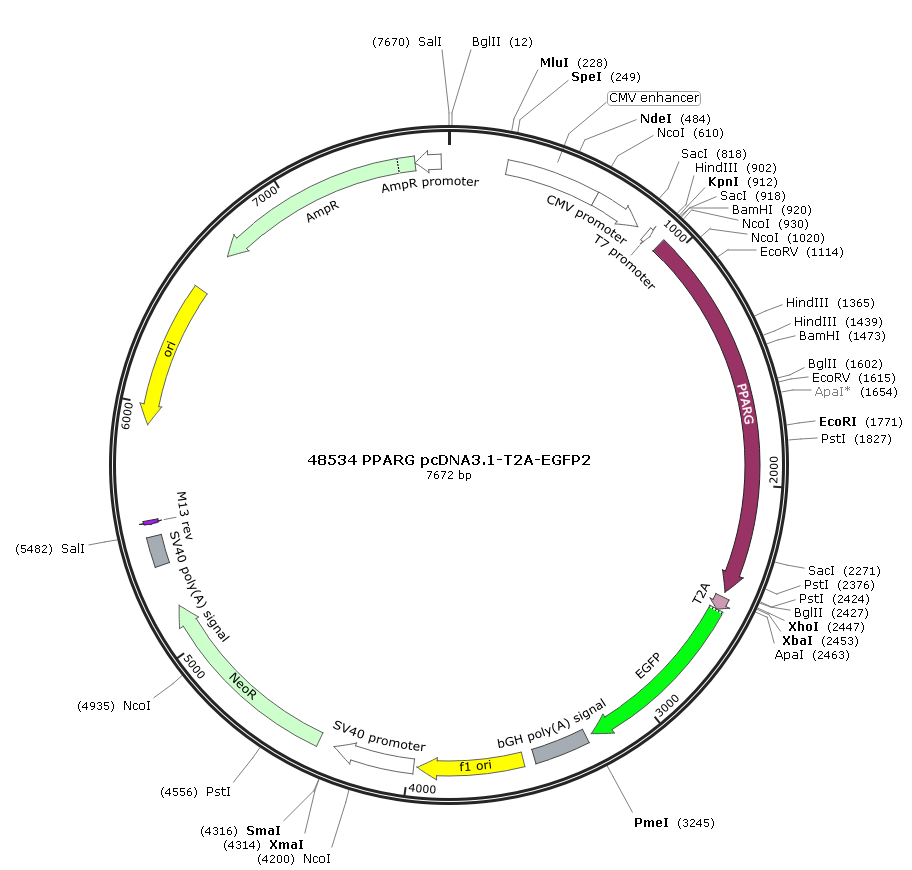


Figure S1: The map of recombinant expression vector of pcDNA 3.1-PPAR-γ (PPARG, Gene ID: 5468). The recombinant plasmid pcDNA 3.1-PPAR-γ was constructed using eukaryotic expression vectors of pcDNA 3.1 and the sequencing results as follow:

GACGGATCGGGAGATCTCCCGATCCCCTATGGTGCACTCTCAGTACAATCTGCTCTGATGCCGCATAGTTAAGCCAGTATCTGCTCCCTGCTTGTGTGTTGGAGGTCGCTGAGTAGTGCGCGAGCAAAATTTAAGCTACAACAAGGCAAGGCTTGACCGACAATTGCATGAAGAATCTGCTTAGGGTTAGGCGTTTTGCGCTGCTTCGCGATGTACGGGCCAGATATACGCGTTGACATTGATTATTGACTAGTTATTAATAGTAATCAATTACGGGGTCATTAGTTCATAGCCCATATATGGAGTTCCGCGTTACATAACTTACGGTAAATGGCCCGCCTGGCTGACCGCCCAACGACCCCCGCCCATTGACGTCAATAATGACGTATGTTCCCATAGTAACGCCAA

S2

TAGGGACTTTCCATTGACGTCAATGGGTGGAGTATTTACGGTAAACTGCCCACTTGGCAGTACATCAAGTGTATCATATGCCAAGTACGCCCCCTATTGACGTCAATGACGGTAAATGGCCCGCCTGGCATTATGCCCAGTACATGACCTTATGGGACTTTCCTACTTGGCAGTACATCTACGTATTAGTCATCGCTATTACCATGGTGATGCGGTTTTGGCAGTACATCAATGGGCGTGGATAGCGGTTTGACTCACGGGGATTTCCAAGTCTCCACCCCATTGACGTCAATGGGAGTTTGTTTTGGCACCAAAATCAACGGGACTTTCCAAAATGTCGTAACAACTCCGCCCCATTGACGCAAATGGGCGGTAGGCGTGTACGGTGGGAGGTCTATATAAGCAGAGCTCTCTGGCTAACTAGAGAACCCACTGCTTACTGGCTTATCGAAATTAATACGACTCACTATAGGGAGACCCAAGCTGGCTAGTTAAGCTTGGTACCGAGCTCGGATCCGCCACCatgggtgaaactctgggagattctcctattgacccagaaagcgattccttcactgatacactgtctgcaaacatatcacaagaaatgaccatggttgacacagagatgc

cattctggcccaccaactttgggatcagctccgtggatctctccgtaatggaagaccactcccactcctttgatatcaagcccttcactactgttgacttctccagcatttctactccacattacgaagacattccattcacaagaacagatccagtggttgcagattacaagtatgacctgaaacttcaagagtaccaaagtgcaatcaaagtggagcctgcatctccaccttattattctgagaagactcagctctacaataagcctcatgaagagccttccaactccctcatggcaattgaatgtcgtgtctgtggagataaagcttctggatttcactatggagttcatgcttgtgaaggatgcaagggtttcttccggagaacaatcagattgaagcttatctatgacagatgtgatcttaactgtcggatccacaaaaaaagtagaaataaatgtcagtactgtcggtttcagaaatgccttgcagtggggatgtctcataatgccatcaggtttgggcggatgccacaggccgagaaggagaagctgttggcggagatctccagtgatatcgaccagctgaatccagagtccgctgacctccgggccctggcaaaacatttgtatgactcatacataaagtccttcccgctgaccaaagcaaaggcgagggcgatcttgacaggaaagacaacagacaaatcaccattcgttatctatgacatgaattccttaatgatgggagaagataaaatcaagttcaaacacatcacccccctgcaggagcagagcaaagaggtggccatccgcatctttcagggctgccagtttcgctccgtggaggctgtgcaggagatcacagagtatgccaaaagcattcctggttttgtaaatcttgacttgaacgaccaagtaactctcctcaaatatggagtccacgagatcatttacacaatgctggcctccttgatgaataaagatggggttctcatatccgagggccaaggcttcatgacaagggagtttctaaagagcctgcgaaagccttttggtgactttatggagcccaagtttgagtttgctgtgaagttcaatgcactggaattagatgacagcgacttggcaatatttattgctgtcattattctcagtggagaccgcccaggtttgctgaatgtgaagcccattgaagacattcaagacaacctgctacaagccctggagctccagctgaagctgaaccaccctgagtcctcacagctgtttgccaagctgctccagaaaatgacagacctcagacagattgtcacggaacacgtgcagctactgcaggtgatcaagaagacggagacagacatgagtcttcacccgctcctgcaggagatctacaaggacttgtacCTCGAGTCTAGAGGGCCCTTCGAGGGCAGAGGAAGTCTTCTAACATGCGGTGACGTGGAGGAGAATCCCGGCCCTATGGTGAGCAAGGGCGAGGAGCTGTTCACCGGGGTGGTGCCCATCCTGGTCGAGCTGGACGGCGACGTAAACGGCCACAAGTTCAGCGTGTCCGGCGAGGGCGAGGGCGATGCCACCTACGGCAAGCTGACCCTGAAGTTCATCTGCACCACCGGCAAGCTGCCCGTGCCCTGGCCCACCCTCGTGACCACCCTGACCTACGGCGTGCAGTGCTTCAGCCGCTACCCCGACCACATGAAGCAGCACGACTTCTTCAAGTCCGCCATGCCCGAAGGCTACGTCCAGGAGCGCACCATCTTCTTCAAGGACGACGGCAACTACAAGACCCGCGCCGAGGTGAAGTTCGAGGGCGACACCCTGGTGAACCGCATCGAGCTGAAGGGCATCGACTTCAAGGAGGACGGCAACATCCTGGGGCACAAGCTGGAGTACAACTACAACAGCCACAACGTCTATATCATGGCCGACAAGCAGAAGAACGGCATCAAGGTGAACTTCAAGATCCGCCACAACATCGAGGACGGCAGCGTGCAGCTCGCCGACCACTACCAGCAGAACACCCCCATCGGCGACGGCCCCGTGCTGCTGCCCGACAACCACTACCTGAGCACCCAGTCCGCCCTGAGCAAAGACCCCAACGAGAAGCGCGATCACATGGTCCTGCTGGAGTTCGTGACCGCCGCCGGGATCACTCTCGGCATGGACGAGCTGTACAAGTAAGTTTAAACCCGCTGATCAGCCTCGACTGTGCCTTCTAGTTGCCAGCCATCTGTTGTTTGCCCCTCCCCCGTGCCTTCCTTGACCCTGGAAGGTGCCACTCCCACTGTCCTTTCCTAATAAAATGAGGAAATTGCATCGCATTGTCTGAGTAGGTGTCATTCTATTCTGGGGGGTGGGGTGGGGCAGGACAGCAAGGGGGAGGATTGGGAAGACAATAGCAGGCATGCTGGGGATGCGGTGGGCTCTATGGCTTCTGAGGCGGAAAGAACCAGCTGGGGCTCTAGGGGGTATCCCCACGCGCCCTGTAGCGGCGCATTAAGCGCGGCGGGTGTGGTGGTTACGCGCAGCGTGACCGCTACACTTGCCAGCGCCCTAGCGCCCGCTCCTTTCGCTTTCTTCCCTTCCTTTCTCGCCACGTTCGCCGGCTTTCCCCGTCAAGCTCTAAATCGGGGGCTCCCTTTAGGGTTCCGATTTAGTGCTTTACGGCACCTCGACCCCAAAAAACTTGATTAGGGTGATGGTTCACGTAGTGGGCCATCGCCCTGATAGACGGTTTTTCGCCCTTTGACGTTGGAGTCCACGTTCTTTAATAGTGGACTCTTGTTCCAAACTGGAACAACACTCAACCCTATCTCGGTCTATTCTTTTGATTTATAAGGGATTTTGCCGATTTCGGCCTATTGGTTAAAAAATGAGCTGATTTAACAAAAATTTAACGCGAATTAATTCTGTGGAATGTGTGTCAGTTAGGGTGTGGAAAGTCCCCAGGCTCCCCAGCAGGCAGAAGTATGCAAAGCATGCATCTCAATTAGTCAGCAACCAGGTGTGGAAAGTCCCCAGGCTCCCCAGCAGGCAGAAGTATGCAAAGCATGCATCTCAATTAGTCAGCAACCATAGTCCCGCCCCTAACTCCGCCCATCCCGCCCCTAACTCCGCCCAGTTCCGCCCATTCTCCGCCCCATGGCTGACTAATTTTTTTTATTTATGCAGAGGCCGAGGCCGCCTCTGCCTCTGAGCTATTCCAGAAGTAGTGAGGAGGCTTTTTTGGAGGCCTAGGCTTTTGCAAAAAGCTCCCGGGAGCTTGTATATCCATTTTCGGATCTGATCAAGAGACAGGATGAGGATCGTTTCGCATGATTGAACAAGATGGATTGCACGCAGGTTCTCCGGCCGCTTGGGTGGAGAGGCTATTCGGCTATGACTGGGCACAACAGACAATCGGCTGCTCTGATGCCGCCGTGTTCCGGCTGTCAGCGCAGGGGCGCCCGGTTCTTTTTGTCAAGACCGACCTGTCCGGTGCCCTGAATGAACTGCAGGACGAGGCAGCGCGGCTATCGTGGCTGGCCACGACGGGCGTTCCTTGCGCAGCTGTGCTCGACGTTGTCACTGAAGCGGGAAGGGACTGGCTGCTATTGGGCGAAGTGCCGGGGCAGGATCTCCTGTCATCTCACCTTGCTCCTGCCGAGAAAGTATCCATCATGGCTGATGCAATGCGGCGGCTGCATACGCTTGATCCGGCTACCTGCCCATTCGACCACCAAGCGAAACATCGCATCGAGCGAGCACGTACTCGGATGGAAGCCGGTCTTGTCGATCAGGATGATCTGGACGAAGAGCATCAGGGGCTCGCGCCAGCCGAACTGTTCGCCAGGCTCAAGGCGCGCATGCCCGACGGCGAGGATCTCGTCGTGACCCATGGCGATGCCTGCTTGCCGAATATCATGGTGG

AAAATGGCCGCTTTTCTGGATTCATCGACTGTGGCCGGCTGGGTGTGGCGGACCGCTATCAGGACATAGCGTTGGCTACCCGTGATATTGCTGAAGAGCTT

GGCGGCGAATGGGCTGACCGCTTCCTCGTGCTTTACGGTATCGCCGCTCCCGATTCGCAGCGCATCGCCTTCTATCGCCTTCTTGACGAGTTCTTCTGAGCS3

GGGACTCTGGGGTTCGCGAAATGACCGACCAAGCGACGCCCAACCTGCCATCACGAGATTTCGATTCCACCGCCGCCTTCTATGAAAGGTTGGGCTTCGGAATCGTTTTCCGGGACGCCGGCTGGATGATCCTCCAGCGCGGGGATCTCATGCTGGAGTTCTTCGCCCACCCCAACTTGTTTATTGCAGCTTATAATGGTTACAAATAAAGCAATAGCATCACAAATTTCACAAATAAAGCATTTTTTTCACTGCATTCTAGTTGTGGTTTGTCCAAACTCATCAATGTATCTTATCATGTCTGTATACCGTCGACCTCTAGCTAGAGCTTGGCGTAATCATGGTCATAGCTGTTTCCTGTGTGAAATTGTTATCCGCTCACAATTCCACACAACATACGAGCCGGAAGCATAAAGTGTAAAGCCTGGGGTGCCTAATGAGTGAGCTAACTCACATTAATTGCGTTGCGCTCACTGCCCGCTTTCCAGTCGGGAAACCTGTCGTGCCAG

CTGCATTAATGAATCGGCCAACGCGCGGGGAGAGGCGGTTTGCGTATTGGGCGCTCTTCCGCTTCCTCGCTCACTGACTCGCTGCGCTCGGTCGTTCGGCTGCGGCGAGCGGTATCAGCTCACTCAAAGGCGGTAATACGGTTATCCACAGAATCAGGGGATAACGCAGGAAAGAACATGTGAGCAAAAGGCCAGCAAAAGGCCAGGAACCGTAAAAAGGCCGCGTTGCTGGCGTTTTTCCATAGGCTCCGCCCCCCTGACGAGCATCACAAAAATCGACGCTCAAGTCAGAGGTGGCGAAACCCGACAGGACTATAAAGATACCAGGCGTTTCCCCCTGGAAGCTCCCTCGTGCGCTCTCCTGTTCCGACCCTGCCGCTTACCGGATACCTGTCCGCCTTTCTCCCTTCGGGAAGCGTGGCGCTTTCTCATAGCTCACGCTGTAGGTATCTCAGTTCGGTGTAGGTCGTTCGCTCCAAGCTGGGCTGTGTGCACGAACCCCCCGTTCAGCCCGACCGCTGCGCCTTATCCGGTAACTATCGTCTTGAGTCCAACCCGGTAAGACACGACTTATCGCCACTGGCAGCAGCCACTGGTAACAGGATTAGCAGAGCGAGGTATGTAGGCGGTGCTACAGAGTTCTTGAAGTGGTGGCCTAACTACGGCTACACTAGAAGAACAGTATTTGGTATCTGCGCTCTGCTGAAGCCAGTTACCTTCGGAAAAAGAGTTGGTAGCTCTTGATCCGGCAAACAAACCACCGCTGGTAGCGGTGGTTTTTTTGTTTGCAAGCAGCAGAT

TACGCGCAGAAAAAAAGGATCTCAAGAAGATCCTTTGATCTTTTCTACGGGGTCTGACGCTCAGTGGAACGAAAACTCACGTTAAGGGATTTTGGTCATGAGATTATCAAAAAGGATCTTCACCTAGATCCTTTTAAATTAAAAATGAAGTTTTAAATCAATCTAAAGTATATATGAGTAAACTTGGTCTGACAGTTACCAATGCTTAATCAGTGAGGCACCTATCTCAGCGATCTGTCTATTTCGTTCATCCATAGTTGCCTGACTCCCCGTCGTGTAGATAACTACGATACGGGAGGGCTTACCATCTGGCCCCAGTGCTGCAATGATACCGCGAGACCCACGCTCACCGGCTCCAGATTTATCAGCAATAAACCAGCCAGCCGGAAGGGCCGAGCGCAGAAGTGGTCCTGCAACTTTATCCGCCTCCATCCAGTCTATTAATTGTTGCCGGGAAGCTAGAGTAAGTAGTTCGCCAGTTAATAGTTTGCGCAACGTTGTTGCCATTGCTACAGGCATCGTGGTGTCACGCTCGTCGTTTGGTATGGCTTCATTCAGCTCCGGTTCCCAACGATCAAGGCGAGTTACATGATCCCCCATGTTGTGCAAAAAAGCGGTTAGCTCCTTCGGTCCTCCGATCGTTGTCAGAAGTAAGTTGGCCGCAGTGTTATCACTCATGGTTATGGCAGCACTGCATAATTCTCTTACTGTCATGCCATCCGTAAGATGCTTTTCTGTGACTGGTGAGTACTCAACCAAGTCATTCTGAGAATAGTGTATGCGGCGACCGAGTTGCTCTTGCCCGGCGTCAATACGGGATAATACCGCGCCACATAGCAGAACTTTAAAAGTGCTCATCATTGGAAAACGTTCTTCGGGGCGAAAACTCTCAAGGATCTTACCGCTGTTGAGATCCAGTTCGATGTAACCCACTCGTGCACCCAACTGATCTTCAGCATCTTTTACTTTCACCAGCGTTTCTGGGTGAGCAAAAACAGGAAGGCAAAATGCCGCAAAAAAGGGAATAAGGGCGACACGGAAATGTTGAATACTCATACTCTTCCTTTTTCAATATTATTGAAGCATTTATCAGGGTTATTGTCTCATGAGCGGATACATA

TTTGAATGTATTTAGAAAAATAAACAAATAGGGGTTCCGCGCACATTTCCCCGAAAAGTGCCACCTGACGTC

The sequencing results of PPAR-γ siRNA as follow: 5’ - AATATGGAGTCCACGAGAT - 3’.

S4

**
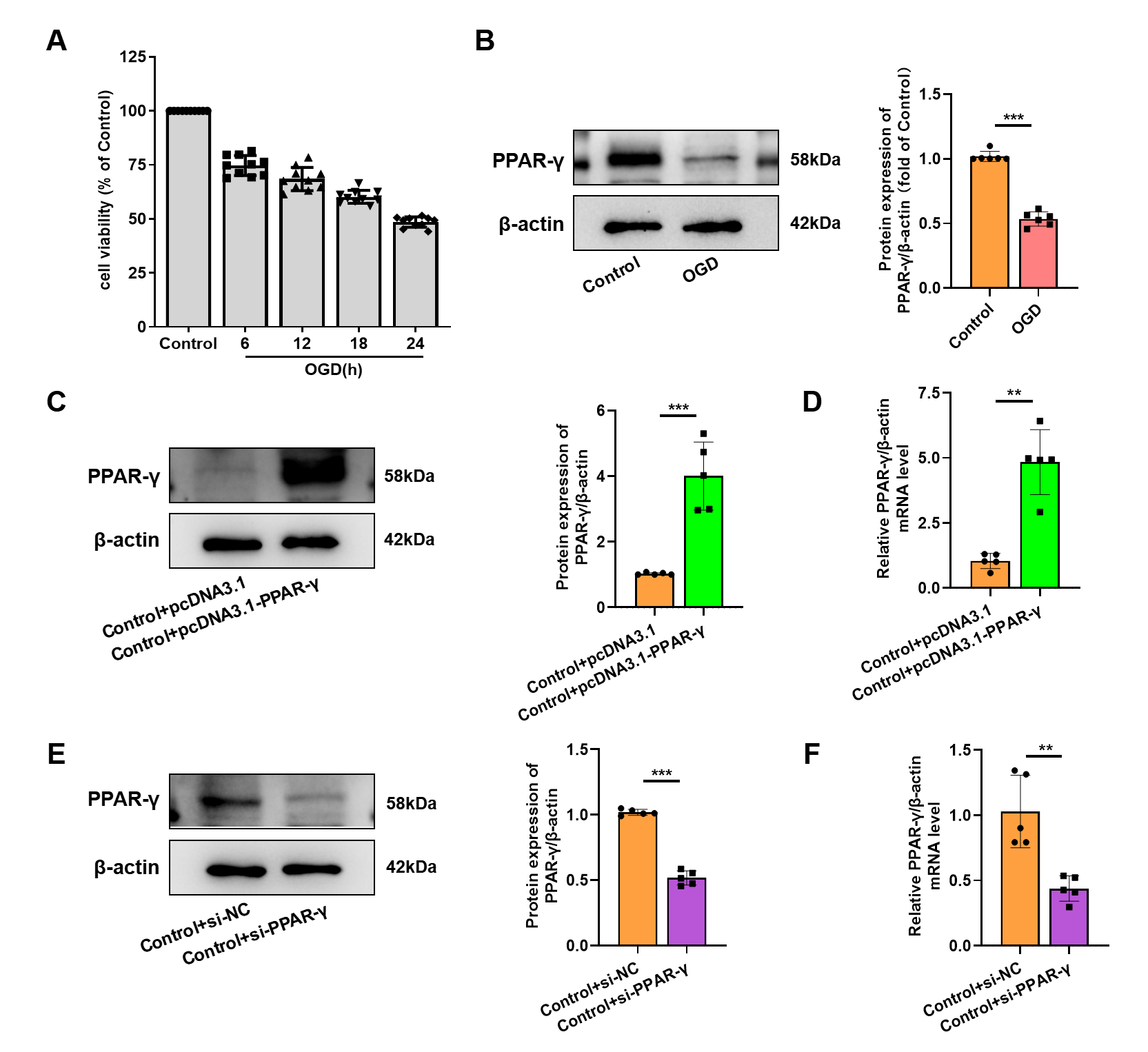
**

**Fig S2. PPAR-γ expression in HMC3 cells after different treatment.**

(A) Effect of OGD on HMC3 cell viability in different time periods was examined using the CCK-8 assay. (n = 10 cultures per condition). (B) Representative images and quantification of western blots for PPAR-γ in HMC3 cells after OGD injury. (C, E) Representative images and quantification of western blots for PPAR-γ in HMC3 cells after pcDNA3.1-PPAR-γor si-PPAR-γ administration. Molecular weight marker (in kDa) is indicated on the right. Data are expressed as fold induction over the control. (D, F) Representative qRT-PCR analysis of PPAR-γexpression levels in HMC3 cells after pcDNA3.1-PPAR-γor si-PPAR-γ administration. Expression levels normalized to β-actin. Graph displays mean ± SD values (n = 5 cultures per condition). **p< 0.01, and ***p< 0.001, significance based on one-way ANOVA with Tukey’s post hoc test.

S5

**Supplementary Table S1.** Primer information for mouse.

| Gene | Primer direction | Sequences(5’→3’) |
| --- | --- | --- |
| NF-κB p65 | Forward | CCTGCTTCTGGAGGGTGATG |
|  | Reverse | GGCTCATACGGTTTCCCATTTA |
| HMGB1 | Forward | GGCGAGCATCCTGGCTTATC |
|  | Reverse | GGCTGCTTGTCATCTGCTG |
| NRF2 | Forward | GCCACCGCCAGGACTACAG |
|  | Reverse | AACTTGTACCGCCTCGTCTGG |
| HO-1 | Forward | AGACCGCCTTCCTGCTCAAC |
|  | Reverse | GACGAAGTGACGCCATCTGTG |
| iNOS | Forward | GTTCTCAGCCCAACAATACAAGA |
|  | Reverse | GTGGACGGGTCGATGTCAC |
| TNF-α | Forward | CCCTCACACTCAGATCATCTTCT |
|  | Reverse | GCTACGACGTGGGCTACAG |
| IL-1β | Forward | GCAACTGTTCCTGAACTCAACT |
|  | Reverse | ATCTTTTGGGGTCCGTCAACT |
| IL6 | Forward | TAGTCCTTCCTACCCCAATTTCC |
|  | Reverse | TTGGTCCTTAGCCACTCCTTC |
| NKX2.2 | Forward | TCTCCAAAGCGCAGACCTAC |
|  | Reverse | GCATCCATCCGTCGGTTTTG |
| SOX10 | Forward | TCCAACCACCCCAAAGACAG |
|  | Reverse | GCAGGTATTGGTCCAGCTCA |
| β-Actin | Forward | GGCTGTATTCCCCTCCATCG |
|  | Reverse | CCAGTTGGTAACAATGCCATGT |

S6

**Supplementary Table S2.** Primer information for human.

S7

| Gene | Primer direction | Sequences(5’→3’) |
| --- | --- | --- |
| NF-κB p65 | Forward | CCTGTCCTTTCTCATCCCATCTTTG |
|  | Reverse | GCTGCCAGAGTTTCGGTTCAC |
| NRF2 | Forward | TTCCTTCAGCAGCATCCTCTCC |
|  | Reverse | AATCTGTGTTGACTGTGGCATCTG |
| HO-1 | Forward | GCCAGTGCCACCAAGTTCAAG |
|  | Reverse | GATGTTGAGCAGGAACGCAGTC |
| iNOS | Forward | TTCAGTATCACAACCTCAGCAAG |
|  | Reverse | TGGACCTGCAAGTTAAAATCCC |
| IL4 | Forward | CGGCAACTTTGTCCACGGA |
|  | Reverse | TCTGTTACGGTCAACTCGGTG |
| CD86 | Forward | CTGCTCATCTATACACGGTTACC |
|  | Reverse | GGAAACGTCGTACAGTTCTGTG |
| CD206 | Forward | GGGTTGCTATCACTCTCTATGC |
|  | Reverse | TTTCTTGTCTGTTGCCGTAGTT |
| IL6 | Forward | ACTCACCTCTTCAGAACGAATTG |
|  | Reverse | CCATCTTTGGAAGGTTCAGGTTG |
| IL-1β | Forward | ATGATGGCTTATTACAGTGGCAA |
|  | Reverse | GTCGGAGATTCGTAGCTGGA |
| TNF-α | Forward | GAGGCCAAGCCCTGGTATG |
|  | Reverse | CGGGCCGATTGATCTCAGC |
| PPAR-γ | Forward | TGAATCCAGAGTCCGCTGACC |
|  | Reverse | CGCCCTCGCCTTTGCTTTG |
| β-Actin | Forward | GGCCAACCGCGAGAAGATGAC |
|  | Reverse | GGATAGCACAGCCTGGATAGCAAC |

**Supplementary Table S3. Primary Antibodies.**

| Antibody | Vendor(city, state, catalogue) | Species | Dilution | |
| --- | --- | --- | --- | --- |
|  |  |  | WB | IHC/IF |
| Olig2 | Proteintech (Wuhan, China, 13999-1-AP) | Rabbit | 1:1000 | 1:300 |
| MBP | Proteintech (Wuhan, China, 10458-1-AP) | Rabbit | 1:1000 | ND |
| MBP | Abacm (Cambridge, MA, USA  Ab218011) | Rabbit | ND | 1:10000 |
| PLP | Affinity Biosciences (Cincinnati, USA, DF13282) | Rabbit | 1:1000 | 1:500 |
| CNPase | Proteintech (Wuhan, China, 13427-1-AP) | Rabbit | 1:1000 | 1:500 |
| MAG | Proteintech (Wuhan, China, 14386-1-AP) | Rabbit | 1:1000 | ND |
| MAG | Santa Cruze (CA, USA, Sc-166849) | Mouse | ND | 1:200 |
| CC1 | Abacm (Cambridge, MA, USA  ab16794) | Mouse | ND | 1:200 |
| PDGFRα | R&D (Minneapolis, MN, USA,  AF1062) | Goat | 1μg/mL | ND |
| NG2 | Proteintech (Wuhan, China, 55027-1-AP ) | Rabbit | 1:1000 | ND |
| PSD95 | Proteintech (Wuhan, China, 20665-1-AP ) | Rabbit | 1:1000 | ND |
| Synaptophysin | Proteintech (Wuhan, China, 17785-1-AP ) | Rabbit | 1:1000 | ND |
| GFAP | Proteintech (Wuhan, China, 16825-1-AP ) | Rabbit | 1:1000 | 1:300 |
| PPAR-γ | Proteintech (Wuhan, China, 16643-1-AP ) | Rabbit | 1:1000 | ND |
| HMGB1 | Proteintech (Wuhan, China, 10829-1-AP ) | Rabbit | 1:1000 | ND |
| p-NF-κB p65 | Affinity Biosciences (Cincinnati, USA, AF2006) | Rabbit | 1:1000 | ND |
| NF-κB p65 | Cell Signalling Technology (Danvers, MA, #8242) | Rabbit | 1:1000 | ND |
| IκBα | Proteintech (Wuhan, China, 10268-1-AP) | Rabbit | 1:1000 | ND |
| p-IκBα | ABclonal (Wuhan, China, AP0707) | Rabbit | 1:1000 | ND |
| Iba1 | Abacm (Cambridge, MA, USA  ab178846) | Rabbit | 1:1000 | 1:500 |
| iNOS | Zenbio (ChengDu, China, 340668) | Rabbit | 1:1000 | 1:500 |
| IL-1β | Zenbio (ChengDu, China, 516288) | Rabbit | 1:1000 | ND |
| IL-1β | Proteintech (Wuhan, China, 16806-1-AP ) | Rabbit | ND | 1:300 |

S8

| SOD2 | Proteintech (Wuhan, China, 24127-1-AP) | Rabbit | 1:1000 | ND |
| --- | --- | --- | --- | --- |
| TNF-α | Wanleibio(Shenyang, China, WL01581) | Rabbit | 1:1000 | ND |
| IL6 | Zenbio (ChengDu, China, 500286) | Rabbit | 1:1000 | ND |
| CD86 | Proteintech (Wuhan, China, 13395-1-AP ) | Rabbit | 1:1000 | ND |
| CD206 | Proteintech (Wuhan, China, 18704-1-AP ) | Rabbit | 1:1000 | ND |
| CD32/CD16 | R&D (Minneapolis, MN, USA,  AF1460) | Goat | 0.1μg/mL | ND |
| CD206 | R&D (Minneapolis, MN, USA,  AF2535) | Goat | 1μg/mL | ND |
| Arginase-1 | Proteintech (Wuhan, China, 66129-1-Ig) | Mouse | 1:1000 | ND |
| NRF2 | Proteintech (Wuhan, China, 16396-1-AP) | Rabbit | 1:1000 | 1:300 |
| KEAP1 | Proteintech (Wuhan, China, 60027-1-Ig) | Mouse | 1:1000 | ND |
| HO-1 | Proteintech (Wuhan, China, 10701-1-AP) | Rabbit | 1:1000 | ND |
| NQO-1 | Proteintech (Wuhan, China, 67240-1-Ig) | Mouse | 1:1000 | ND |
| SOD2 | Proteintech (Wuhan, China, 24127-1-AP) | Rabbit | 1:1000 | ND |
| ZO-1 | Affinity Biosciences (Cincinnati, USA, AF5145) | Rabbit | 1:1000 | ND |
| p120 Catenin | Proteintech (Wuhan, China, 12180-1-AP) | Rabbit | 1:1000 | ND |
| β-Catenin | Proteintech (Wuhan, China, 51067-2-AP) | Rabbit | 1:1000 | ND |
| Claudin-5 | Bioworld Technology (Nanjing, China, BS1069) | Rabbit | 1:1000 | ND |
| Occludin | Abacm (Cambridge, MA, USA  ab216327) | Rabbit | 1:1000 | ND |
| Lamin B | Proteintech (Wuhan, China, 12987-1-AP) | Rabbit | 1:10000 | ND |
| β-actin | Affinity Biosciences (Cincinnati, USA, AF7018) | Mouse | 1:10000 | ND |

Abbreviations: IF, immunofluorescence; IHC, immunohistochemistry; ND, not detected; WB, western blot.

S9
